# Surface-active ionic liquids in micellar catalysis: impact of anion selection on reaction rates in nucleophilic substitutions†
†Electronic supplementary information (ESI) available: Formulae for calculating aggregation parameters and fitting of kinetic constants and copies of NMR spectra. See DOI: 10.1039/c6cp00493h



**DOI:** 10.1039/c6cp00493h

**Published:** 2016-04-28

**Authors:** Alice Cognigni, Peter Gaertner, Ronald Zirbs, Herwig Peterlik, Katharina Prochazka, Christian Schröder, Katharina Bica

**Affiliations:** a Institute of Applied Synthetic Chemistry , Vienna University of Technology , Getreidemarkt 9/163 , 1060 Vienna , Austria . Email: katharina.schroeder@tuwien.ac.at ; Fax: +43 1 58801 16360 ; Tel: +43 1 58801 163601; b Group for Biologically Inspired Materials , Institute of Nanobiotechnology (DNBT) , University of Natural Resources and Life Sciences , Muthgasse 11 , 1190 Vienna , Austria; c Dynamics of Condensed Systems , University of Vienna , Boltzmanngasse 5 , 1090 Vienna , Austria; d Institute of Computational Biological Chemistry , University of Vienna , Währingerstrasse 17 , 1090 Vienna , Austria

## Abstract

A series of surface-active ionic liquids based on the 1-dodecyl-3-methylimidazolium cation and different anions such as halides and alkylsulfates was synthesized. The aggregation behavior of these ionic liquids in water was characterized by surface tension, conductivity measurements and UV-Vis spectroscopy in order to determine the critical micelle concentration (CMC) and to provide aggregation parameters. The determination of surface activity and aggregation properties of amphiphilic ionic liquids was accompanied by SAXS studies on selected surface-active ionic liquids. The application of these surface-active ionic liquids with different anions was tested in nucleophilic substitution reactions for the degradation of organophosphorus compounds. Kinetic studies *via* UV-Vis spectrophotometry showed a strong acceleration of the reaction in the micellar system compared to pure water. In addition, an influence of the anion was observed, resulting in a correlation between the anion binding to the micelle and the reaction rate constants, indicating that the careful choice of the surface-active ionic liquid can considerably affect the outcome of reactions.

## Abstract 2

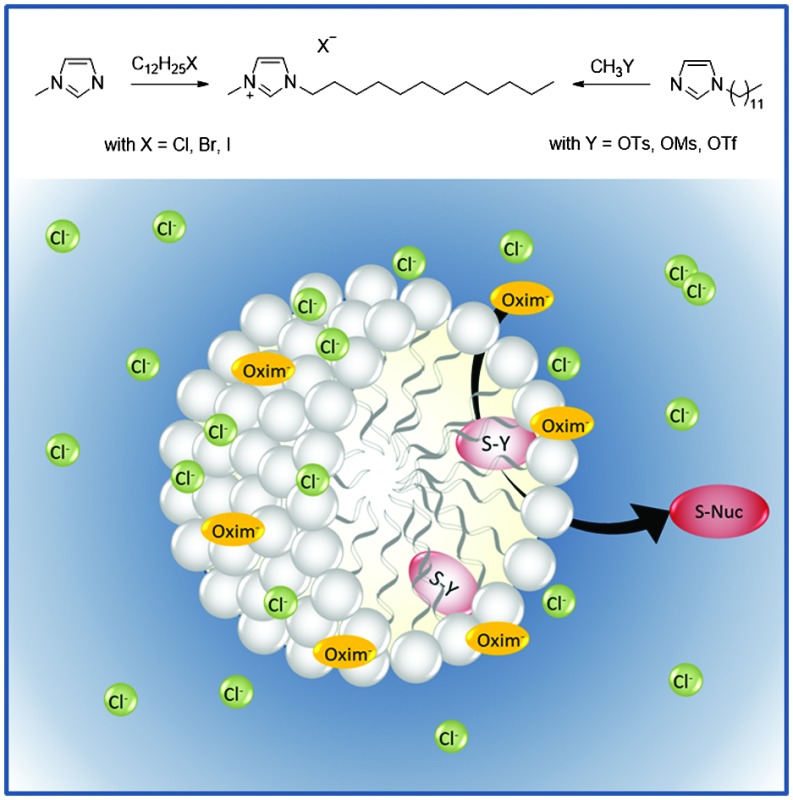
A series of surface-active ionic liquids based on the 1-dodecyl-3-methylimidazolium cation and different anions was synthesized and applied for micellar catalysis of nucleophilic substitutions.

## Introduction

In recent years the scope of ionic liquids as functional fluids has been expanded to include their mixtures with water for multiple applications.^1^ The microstructure and heterogeneity in the liquid state, fundamental aspects of pure ionic liquids, continue to exist in aqueous solution, and several reviews have been published focusing on the structure and behaviour of ionic liquid clusters in water.^2^ The self-assembly of ionic liquids in aqueous solution is particularly well investigated for long alkyl chain ionic liquids that have been found to display amphiphilic character analogous to conventional cationic surfactants.^3^ The co-existence of an ionic hydrophilic head group and a long hydrophobic tail results in the formation of aggregates in water; however, as a general rule the alkyl chain must contain at least eight carbon atoms for aggregation in water.^4^ A number of computational and experimental techniques, including molecular dynamics simulations, surface tension and conductivity measurements, potentiometry, UV-Vis spectroscopy, fluorescence probes, NMR spectroscopy, mass spectrometry, isothermal titration calorimetry, light scattering and small-angle X-ray and neutron scattering (SAXS and SANS) have been applied to understand the self-aggregation of surface-active ionic liquids in water.^4–11^

While traces of water in ionic liquids are often considered as problematic due to the difficulty in their removal and their large impact on physical properties, the deliberate combination of these two media in well-defined mixtures may result in unique properties that can be fully exploited by varying their composition.^12^ For example, the limited solubility of many organic compounds in water could be enhanced with ionic liquids, whereas price or viscosity issues of ionic liquids could be balanced when used in aqueous solution. At the same time the specific properties of the highly tunable ionic liquids and the strong hydrogen bond of water can be combined to afford a reaction environment with new properties. Applications of well-defined ionic liquid/water mixtures have already been identified and range from biomolecule stabilization towards their application in catalysis or in novel separation processes. The impact of self-assembly of ionic liquids in aqueous solution has also been exploited by our group, showing that surface-active ionic liquids can be efficiently applied in micellar catalysis of Diels Alder reactions, for asymmetric transfer hydrogenations in water, and also for extraction of natural products.^13,14^

Studies on the self-assembly and structural organization of ionic liquids in water have so far dealt mainly with monocationic alkyl imidazolium based ionic liquids, however also other structures have already been investigated. Full critical micellar concentration (CMC) characterization and extensive thermodynamic studies allow identifying specific features that influence the properties of these ionic liquids. Several studies showed that the CMC is strongly influenced by the structure of the ionic liquid, in particular in terms of the alkyl chain length of the hydrophobic tail unit.^3–6,15–19^ As for traditional cationic surfactants, the Klevens equation was found to be valid.^20^ A linear relationship between the number of carbon atoms on the alkyl chain and the logarithm of the CMC has been verified^19^ which can be applied for predicting the CMC for a homologous series of linear-single chain amphiphiles at a fixed temperature. Cations with different structures have also been reported.^5,6,21^ Blesic *et al.* ^21^ investigated the effect of different cationic rings by comparing alkylmethylimidazolium with alkylmethylpyridinium, alkylmethylpiperidinium and alkylmethylpyrrolidinium core groups. The results show that pyrrolidinium has the lowest CMC while the others present an almost identical surface tension profile, however due to the very complex interactions a univocal explanation based on geometry and/or electronic distribution could not be provided. This is in accordance with a study of Cornellas *et al.* ^5^ where no substantial difference was identified in the behaviour for surface-active ionic liquids with imidazolium and pyridinium head groups. The introduction of an ester or amide functionality in the side chain of these head groups resulted in improved surface activity according to the studies of Comelles *et al.* ^22,23^; moreover biodegradability was improved by the ester functionality, while the amide group provided a higher thermal stability. On the other hand El Seoud *et al.* ^24^ showed that the introduction of a vinyl moiety on the imidazolium cation resulted in higher CMC values compared to the corresponding ethyl analogues due to the less hydrophobic character of the vinyl group. Baltazar *et al.* ^25^ also investigated the aggregation behaviour of dicationic alkylimidazolium ionic liquids, and CMC values were found to depend not only on the chain length of the imidazolium cation, but also on the length of the linkage chain between the two head groups. Similar results were also obtained by Anderson *et al.* ^26^ who investigated tricationic surface active ionic liquids. Besides showing a better surface activity compared to dicationic ones, this could be further improved by increasing the size and aromaticity of the internal linker. In addition, surface-active ionic liquids with different anions have been investigated, but studies on the role of the anion are less systematic. Blesic *et al.* ^4^ compared the aggregation behaviour of 1-dodecycl-3-methylimidazolium ([C_12_mim]^+^) based ionic liquids with Cl^–^, [PF_6_]^–^, and [NTf_2_]^–^ as counterions. Aggregates in aqueous solution were found only in the case of chloride anions, whereas the low solubility of the ionic liquids containing the [PF_6_]^–^ and [NTf_2_]^–^ anions resulted in phase separation prior to the formation of aggregates. In a different study on different halide counterions Kim *et al.* reported higher surface activity for [C_12_mim]I than for the same ionic liquids containing Br^–^ or Cl^–^ as a counter anion; this was explained by the higher polarizability and binding to the micelles of iodide ions.^27^

In a study conducted by Frizzo *et al.* ^28^ concerning dicationic imidazolium based surface-active ionic liquids dissolved in 4.75% ethanol–water solution, a correlation between the aggregation and the anion hydrophobicity was identified. Recently Nowicki *et al.* ^29^ reported a series of alkylimidazolium surface-active ionic liquids with HSO_4_^–^ as a counterion. These ionic liquids had a key role as co-catalysts in an oxirane ring opening reaction of epoxidized methyl oleate, and their performance was dependent on the length of the alkyl chain attached to the imidazolium cation. Using a bulky amphiphilic anion such as ibuprofenate allows detection of a critical aggregation concentration even when coupled with [C_4_mim]^+^ or [C_6_mim]^+^ cations; moreover it was suggested that ionic liquids containing an active pharmaceutical ingredient can be incorporated into ionogels and used for drug delivery applications.^30^

In contrast, fewer studies exist on ionic liquids with surface-active anions in the literature. Early examples report surface-activity of octylsulfate combined with [C_4_mim]^+^, which also contributes to a decrease in the toxicity of the ionic liquid.^10,31^ Comprehensive work was conducted by Blesic *et al.* ^32,33^ on alkylimidazolium alkylsulfonate ionic liquids. A matrix of ionic liquids with variable chain length in both the cation and the anion was synthesized and the physico-chemical properties were determined. Again, a strong dependence of the CMC on the chain length of the cation was identified, but the surface activity could be considerably improved by simultaneously introducing a long alkyl chain in the anion. A series of protic ionic liquids based on long-alkyl chain carboxylates was investigated with different cations such as imidazolium and pyrrolidinium. These ionic liquids not only showed a higher surface activity than cationic surfactants, but also performed better than the corresponding surfactants with inorganic cations.^34^ When diisopropylethylammonium was used as a cation, the formation of bilayer vesicle-like aggregates was also demonstrated.^35^

Eventually, fluorinated surface-active ionic liquids composed of perfluorinated anions have only recently been investigated.^36,37^ An extensive study by Pereiro *et al.* showed that perfluorinated alkylsulfonates strongly decrease the CMC in comparison to the corresponding halide-based ionic liquids, and even for [C_2_mim][C_4_F_9_SO_3_] and [C_2_mim][C_8_F_17_SO_3_] a critical aggregation concentration could be detected.

Herein, we intend to present further insights into the nature of self-organization of ionic liquids in water and its impact on micellar catalysis of nucleophilic substitution reactions in water. In particular, we focused on the role of the counteranion and aimed for a correlation between anion binding to the micelle and reaction rate constants, suggesting that the careful choice of the surface-active ionic liquid can considerably affect the outcome of reactions.

## Results and discussion

### Synthesis of surface-active ionic liquids

**Scheme 1:**
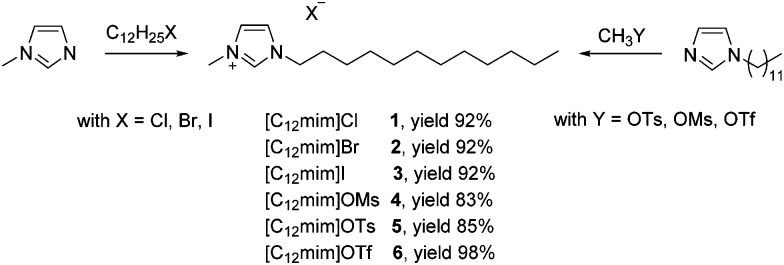
Synthesis with surface-active ionic liquids based on the 1-dodecycl-3-methylimidazolium cation with variable anions.

We focused on 1-dodecycl-3-methylimidazolium-based ionic liquid [C_12_mim]X, as this is a known and already characterized surface-active cation and we functionalized it with different anions. Our synthetic route is based on two different approaches (Scheme 1). In the case of the surface active [C_12_mim]Cl **1**, [C_12_mim]Br **2** and [C_12_mim]I **3** the classical alkylation of *N*-methylimidazole with the corresponding dodecyl halides was performed. In contrast, surface-active ionic liquids [C_12_mim]OMs **4** and [C_12_mim]OTs **5** and [C_12_mim]OTf **6** were prepared in a two-step synthesis route, including the initial synthesis of *N*-dodecylimidazole by alkylation of imidazole. Afterwards the final ionic liquids were obtained *via* direct alkylation of *N*-dodecylimidazole with the methyl esters of the corresponding anions. This second route offers a halide free pathway for the synthesis of ionic liquids. In any case, good yields between 83 and 98% were obtained. The final products were purified by repeated crystallization from organic solvents to isolate all ionic liquids as colourless crystals in high purity. In contrast, crystallization was not possible for [C_12_mim]OTf **6**; however, the highly volatile anion precursors could be easily removed under reduced pressure, allowing isolation of the ionic liquid as a low-melting colourless solid.

### Characterization of surface-activity

Prior to their application in micellar catalysis all ionic liquids were characterized by different experimental techniques in order to investigate the formation of aggregates in water. Several techniques can and have been used for the determination of the CMC of alkyl methyl imidazolium-based ionic liquids, and the reported literature values for a specific ionic liquid vary considerably according to the different techniques as can be seen from the ionic liquid [C_14_mim]Cl, where CMC values ranging from 2.98–4.6 mM have been reported. Herein, we selected three different techniques, including surface tension, conductivity and UV-Vis spectroscopy. The results are summarized in Table 1 and will be discussed in the following paragraphs in detail.

**Table 1:** Physical-chemical characterization of the surface-active ionic liquids

Ionic liquid^*a*^	Surface tension^*b*^				Conductivity^*c*^			UV-Vis^*d*^
	CMC (mM)	*Π*_CMC_ (mN m^–1^)	*A*_min_ (nm^2^)	*P*	CMC (mM)	*β*	Δ*G*0mic (kJ mol^–1^)	CMC (mM)
[C_12_mim]Cl **1**	13.25 (Lit. 13.17)^6^	28.6	0.72	0.29	14.53	0.43	–29.1	—
[C_12_mim]Br **2**	9.29	29.3	0.71	0.30	10.29	0.70	–36.2	10.24
[C_12_mim]I **3**	4.76	37.7	0.62	0.34	5.19	0.84	–42.3	4.12
[C_12_mim]OMs **4**	12.50	29.4	1.13	0.18	14.71	0.45	–29.5	15.26
[C_12_mim]OTf **5**	2.37	40.2	0.55	0.38	3.31	0.73	–41.7	2.69
[C_12_mim]OTs **6**	2.25	38.3	0.65	0.32	3.44	0.89	–45.4	—

^*a*^Solutions were prepared with doubly-distilled Millipore Milli-Q water and left under shaking with 360 min^–1^ for 24 h to equilibrate. Samples were equilibrated at 25.0 ± 0.1 °C using a HAAKE K15 thermostat before measurements.

^*b*^Surface tension was determined using the Du Noüy ring method on a Krüss tensiometer.

^*c*^Conductivity measurement were performed at 25 °C using a Mettler Toledo SevenExcellence InLAB® 741-ISM electrode.

^*d*^UV-Vis measurements were performed at 25 °C, following the absorbance at 312 nm using benzoylacetone. *Π*_CMC_ is the effectiveness of the surface tension reduction, *A*_min_ is the area per molecule residing at the surface, *P* is the packing parameter, *β* is the degree of counterion binding, Δ*G*0mic is Gibbs energy of micellization (for details see the ESI).

### Conductivity measurements

**Fig. 1:**
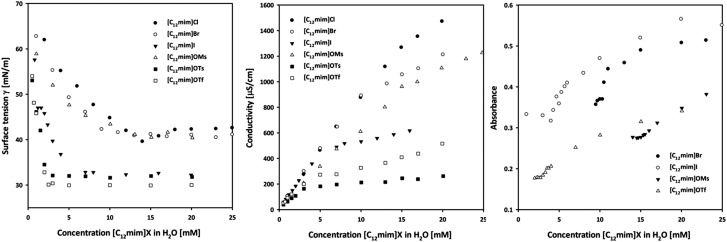
Surface tension (left), conductivity (center) and UV-Vis profiles (right) of the investigated ionic liquids at 25 °C.

Conductivity measurements can be utilized for the characterization of ionic surfactants due to the different mobility of the individual ions and their aggregates. Curves of the specific conductivity (*κ*) *versus* the ionic liquid concentration are reported in Fig. 1 (left). Two different linear regimes could be typically identified that allowed calculating the CMC values *via* their breaking point. In principle, two phenomena are responsible for this curve shape: formed micelles have a lower mobility than single ions. Moreover, parts of the anions are adsorbed on the surface of the aggregates, decreasing the total species contributing to the solution conductivity upon formation of micelles. The degree of counterion binding (*β*) can be estimated from the ratio of the slopes^27^ and indicates the amount of anions on the surface of the micelles. The observed CMC values for ionic liquids already reported are in agreement with the results obtained by other research groups.^27,32^ A clear trend can be seen in the halide series, and CMC values decrease in the order Cl > Br > I. Similarly, a trend is visible for sulfonates where tosylate (OTs^–^) and triflate (OTf^–^) based ionic liquids have a lower CMC than [C_12_mim]OMs **4**. A larger and more hydrophobic anion such as OTs^–^ tends to adsorb on the micellar surface instead of being hydrated, and consequently favours the formation of aggregates by shielding the electrostatic repulsion forces of the charged headgroups.^38^ This behaviour is also supported by the degree of counterion binding (*β*): a higher value corresponds to a lower CMC due to the higher amount of anions bound to the micellar surface. Additionally, the Gibbs energy of micellization (Δ*G*0mic) could be calculated from the conductivity data, proving that the formation of micelles is a spontaneous process that is accompanied by a negative free energy change (see the ESI† for details).^39^

### Surface-tension measurements

Fig. 1 (center) displays the surface tension (*γ*) curves as a function of the concentration of ionic liquids. As a consequence of absorption of ionic liquids at the surface, the surface tension decreases with increasing ionic liquid concentration, until a stable value is obtained after surface saturation. The breaking point of the curve can be used to determine the CMC value of the respective surfactant (see the ESI† for details). This characteristic behaviour allowed calculating CMC values for all ionic liquids. In general, the obtained CMC values were systematically slightly lower than those measured *via* the conductivity, however the same trend can be observed for the anions. According to the effectiveness of the surface tension reduction (*Π*_CMC_) all investigated ionic liquids reduce the water surface tension of 30–40 mN m^–1^. The effectiveness can also be related to the degree of packing of adsorbed surfactant molecules at the interface. The Gibbs adsorption isotherm equation can be used to estimate the average area per molecule residing at the surface (*A*_min_), assuming that the structure pattern corresponds to a monolayer.^40^ A lower *A*_min_ value corresponds to a closer packing of monomers at the interface. This value could be used to estimate the shape of the formed aggregates according to the packing parameter^41^ (*P*) which includes the volume (*v*) of the hydrocarbon chain embedded in the hydrocarbon core of the aggregate and the maximum effective length (*l*_c_) that the chain can assume, calculated according to the Tanford equation (see the ESI† for details).^42^ In general, a packing parameter *P* < 0.33 corresponds to spherical micelles, an intermediate value of 0.33 < *P* < 0.5 corresponds to non-spherical, ellipsoidal micelles, while higher values correspond to rod-like micelles and various interconnected structures.^41^ The results in Table 1 show that both spherical and ellipsoidal aggregates are presumably formed and depend on the anion size. According to this methodology, smaller anions such as bromide or mesylate form spherical micelles, while bigger anions, *e.g.* iodide or tosylate, favor the formation of ellipsoidal aggregates. This is to some extent in agreement with a study on the shape of alkyl imidazolium bromides by Goodchild *et al.*,^9^ who performed SANS experiments and found that [C_12_mim]Br forms spherical particles at low concentration near the CMC, while the shape tends to turn into elongated micelles at higher concentrations.

### UV-Vis measurements

The determination of the CMC of surfactants using UV-Vis spectroscopy is based on the ketoenolic equilibrium of benzoylacetone. The enolic form can be stabilized by intramolecular hydrogen bonding forming a 6-membered ring. This structure is favoured inside the micelles where no competition for hydrogen bonding with the solvent is present. Consequently the formation of micelles with increasing ionic liquid concentration will shift the ketoenolic equilibrium towards the enol form. This results in an increase of the absorbance at 312 nm which can be used to calculate the CMC value from the breaking point of the curves.^43,44^ The values depicted in Fig. 1 (right) are in good agreement with those obtained using the previous techniques, with the exception of [C_12_mim]Cl **1**, where we were not able to find an unambiguous break point. However, we could not perform measurements for [C_12_mim]OTs due to the overlap of the absorption spectrum of the ionic liquids with that of benzoylacetone.

### Small-angle X-ray scattering

The determination of the surface activity and aggregation properties of amphiphilic ionic liquids was accompanied by SAXS studies. As the SAXS measurements and the evaluation were more time consuming, we decided to select three representative ionic liquids **1**, **2** and **6**, which cover the observed range of different conductivities (high, medium, and low conductivity in Fig. 1, center).

**Fig. 2:**
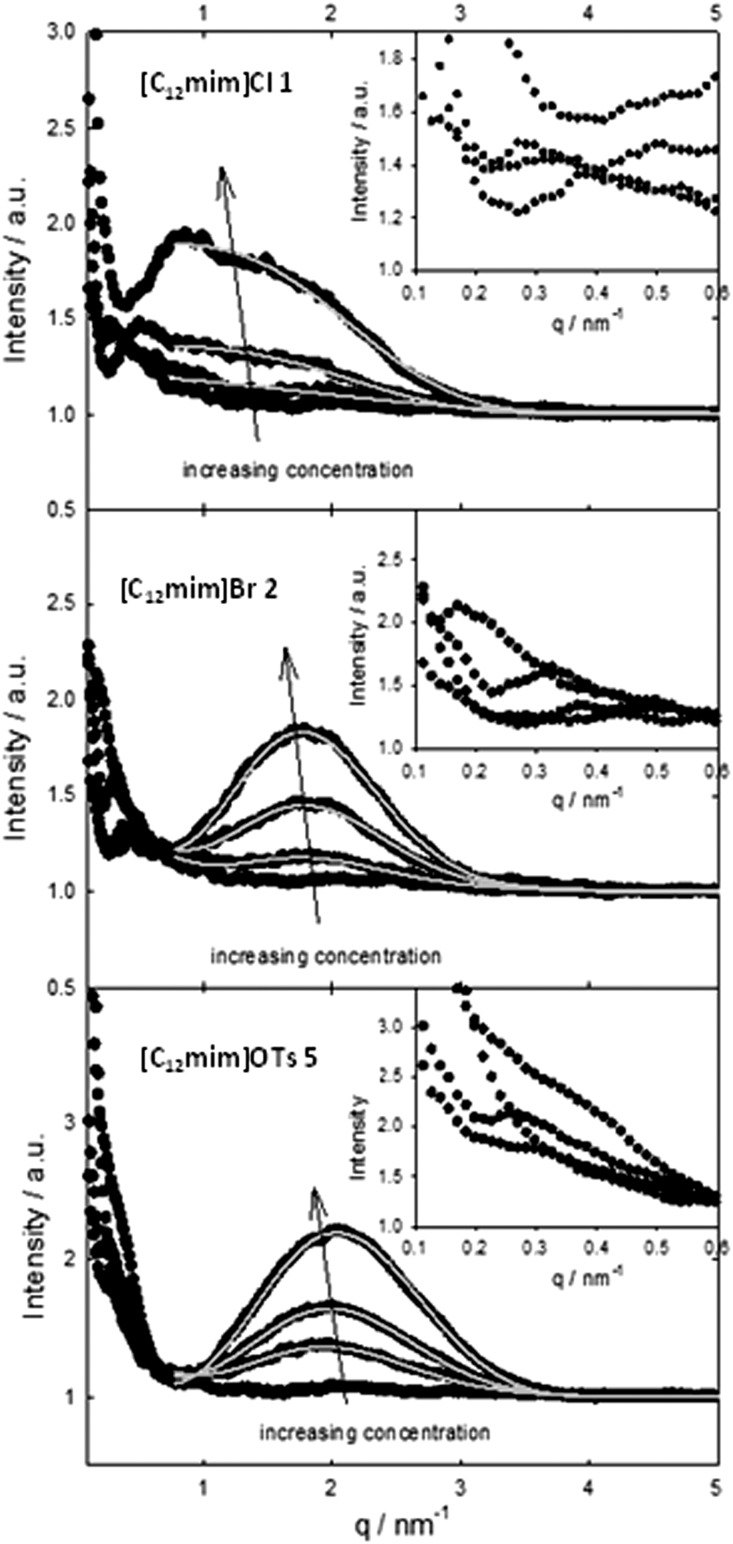
Small-angle X-ray intensities for the different ionic liquids. The experimental intensities are shown as filled circles, the fit from the core–shell model with grey fit lines and the insets show the scattering intensities towards very low *q*-values.

The SAXS intensities (Fig. 2) were fitted using a model describing spherical micelles with a radius of the core *R*_c_, a Schulz distribution for the radii and a shell thickness *δ*. The core radius is about 1 nm and exhibits a more uniform and narrow size distribution with increasing concentration (see Fig. S1a to c in the ESI†). The shell has a similar size, about 1 nm for [C_12_mim]Cl **1** and slightly higher for [C_12_mim]Br **2** and [C_12_mim]OTs **6**. The most important information is the parameter *γ*, which describes the electron differences between the core, shell and surrounding medium^45^ and is defined in the ESI:† it decreases with increasing concentration as well as it is significantly lower for [C_12_mim]Cl (even close to zero for [C_12_mim]Cl at 100 mM concentration) than for both other ionic liquids. As a value of *γ* = 0 means that the scattering only arises from the core^45^ and the density of the shell is the same as the density of the surrounding solution, one may conclude that Cl is mobile and distributed in the solution, whereas OTs is only very weakly affected by different concentrations (Table 2). The inset in Fig. 2 shows the scattering intensities towards very low *q*-values. A weak maximum is also visible, which shifts towards smaller *q*-values for higher concentrations. This can be interpreted as a weak tendency for agglomeration of micelles with a cluster size of approximately 15 nm for the 25 mM concentration and considerably larger for higher concentration.

**Table 2:** Fit parameters for spheres with a core shell structure and a Schulz distribution of the radius of the core (parameters core radius *R*_c_, *Z*). *γ* is the scaled medium contrast (*γ* = 1, the scatter origins only from the shell, *γ* = 0, only from the core)^35^

Ionic liquid	Concentration/mM	Core radius *R*_c_/nm	*Z*	Shell thickness *δ*/nm	*γ*
[C_12_mim]OTs	25	0.95	15	1.23	0.16
[C_12_mim]OTs	50	0.97	28	1.26	0.13
[C_12_mim]OTs	100	1.02	84	1.22	0.12
[C_12_mim]Br	25	0.97	8.3	1.27	0.20
[C_12_mim]Br	50	0.97	13	1.39	0.13
[C_12_mim]Br	100	0.98	25	1.59	0.09
[C_12_mim]Cl	25	1.14	52	0.95	0.07
[C_12_mim]Cl	50	1.18	250	0.94	0.06
[C_12_mim]Cl	100	1.14	51	0.94	0.007

### Application in nucleophilic substitutions

Micellar catalysis has already been proven to be a very powerful tool to overcome issues of conventional synthesis in both pure organic solvents and water, and recent developments in the fields have been thoroughly reviewed.^46–48^ The problems created by the massive use of volatile, toxic and flammable organic solvents in industrial processes have increased the demand for more benign reaction media, and water is a prime candidate here. However, issues related to the poor solubility of organic compounds in water are well known, a problem that can be overcome *via* the addition of surfactants. It has already been reported that surface active ionic liquid aggregates can increase the solubility of apolar organic compounds that exhibit very poor solubility in water.^49^ Furthermore micelles have the capability to drastically alter reaction rates of organic reactions, as the local concentration experienced by the reactants at/in the micellar zone is higher than in a bulk solution. Surfactants based on ionic liquids can also be finely tuned by adjustments on the head group, alkyl chain or counterion structure in order to offer an optimal reaction environment increasing parameters such as yield, rate and selectivity. Successful results were already obtained in our previous research on Diels–Alder reactions.^14^ The highest reaction rate occurred above the CMC, clearly showing the positive effect of ionic liquid-derived micelles.

Nucleophilic substitutions of organophosphorus compounds can be accelerated by cationic surfactants and are strongly influenced by different anions present in the reaction mixture.^50^ Optimal conditions for the substitution of organophosphorus compounds are obtained with α-nucleophiles, *i.e.* nucleophiles possessing a heteroatom with an unshared electron pair adjacent to the nucleophilic centre that exhibit a higher nucleophilic reactivity compared with common nucleophiles of similar basicity, a concept that is also known as the “α-effect”. More specifically, oximates have been shown to efficiently promote the hydrolysis of phosphate triesters as they prevent the competition with other nucleophiles such as OH^–^ or halides.^51–53^ Studies on the effect of several oximes^54^ and substrate features^51,53^ have been reported.

**Scheme 2:**
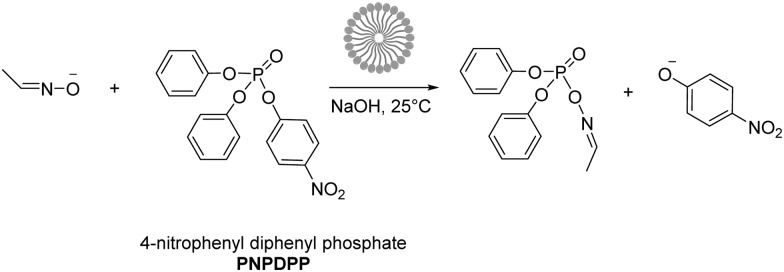
Nucleophilic substitution of 4-nitrophenyl diphenyl phosphate (PNPDPP) with acetaldoxime.

In this study, we investigated the reaction between 4-nitrophenyl diphenyl phosphate (PNPDPP) and acetaldoxime (p*K*_a_ = 11.82) in different surface-active ionic liquid/water mixtures (Scheme 2). PNPDPP is a highly lipophilic substrate which tends to interact strongly with micellar aggregates in aqueous solution. Moreover, it has an analogous structure to pesticides such as paraoxon or fenitron, both being toxic organophosphorous compounds that are widely used as pesticides in agriculture for field crop and fruit protection against a variety of insects and show considerable toxicity towards human and other mammals.^54^ In the literature second order kinetic constants of the reaction occurring in the micellar phase and in the bulk water are reported to be very similar, consequently, we were only interested in the observed pseudo first order kinetic constant that shows the overall reaction rate acceleration.^53,54^ The substitution reaction was conducted under pseudo-first order conditions where the oxime was used in 100-fold excess – an oxime concentration high enough to act as a buffer itself. Different possible reaction pathways have been reported for nucleophilic substitutions of organophosphorus compounds. In our case the only possible pathway is the attack of the nucleophile at the phosphorous centre, which follows a S_N_2(P) mechanism,^54^ while the attack on the aromatic carbons is very unlikely.^53,55,56^ In principle, two leaving groups are possible, either the favored *p*-nitrophenolate, whose formation can be observed at 402.5 nm or phenolate, which cannot be seen due to the spectral overlap with other components in the reaction mixture. We observed that the final absorbance reached during the kinetic runs did not correspond to the maximum theoretical one, suggesting the formation of a by-product. This was verified through ^31^P experiments, where the formation of both *p*-nitrophenyl phenyl phosphate and diphenyl phosphate as by-products was observed, although the formation of the latter one was considerably slower. Eventually, rate constants for both the desired reaction and the by-product formation were calculated through the least-squares exponential fit of the experimental data: results reported here as observed reaction rates *k*_obs_ refer to the desired nucleophilic reaction only (see the ESI† for details).

**Fig. 3:**
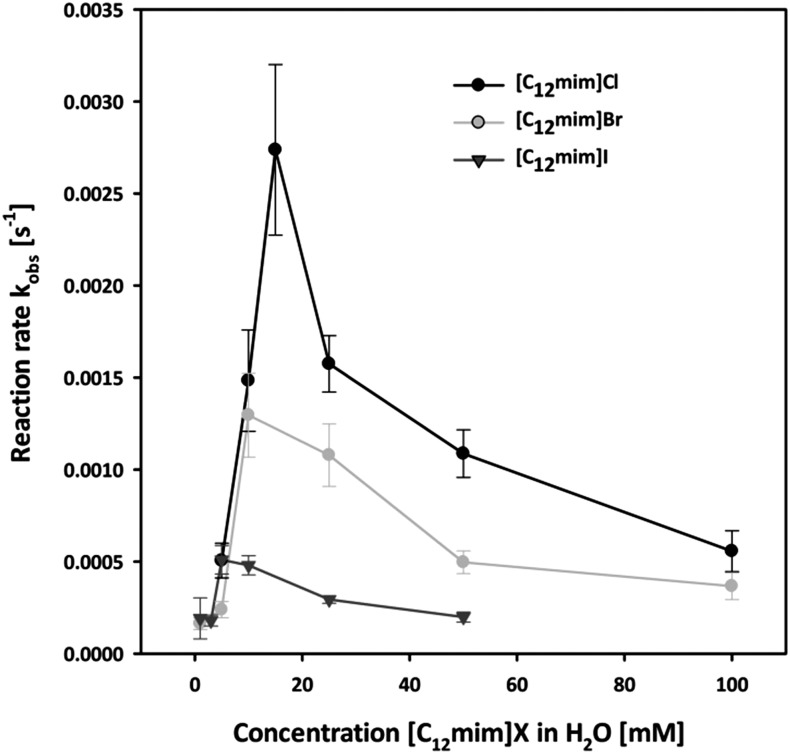
Pseudo first order reaction rate constant of the nucleophilic substitution of nitrophenyl diphenyl phosphate (PNPDPP) with acetaldoxime as a function of [C_12_mim]X with X = Cl, Br and I in water.

In order to investigate the effect of our IL anions we initially investigated the reaction in all halide based ionic liquids [C_12_mim]X with X = Cl, Br and I in the concentration range from 0 to 100 mM. The observed reaction rates are shown in Fig. 3, and three interesting factors can be identified: in all cases a strong increase in the reaction rate was observed with increasing concentration of ionic liquids, resulting in strongly enhanced reaction rates compared to pure water. This rate enhancement in the micellar medium arises from two effects: on the one side the lipophilic substrate accumulates in the micelles, and at the same time the electrostatic attraction of the cationic head groups to the negatively charged nucleophile leads to the increment of its concentration at the micellar interface where the reaction takes place.

Moreover, it can be observed that in all three cases the maximum acceleration for each ionic liquid corresponds to their CMC. Below the CMC the formation of aggregates induced by the presence of the solute causes the rate enhancement compared to water, while above the CMC most probably both a dilution effect over more micelles and a higher competition with the higher amount of inert anions take place. This effect is in accordance with the concept of micellar catalysis and has also been observed in our previous work on Diels–Alder reaction in surface-active ionic liquid/water mixtures.^14^

Most importantly, the highest enhancement of the reaction rate was observed with chloride-based ionic liquids, suggesting that the choice of anion plays a fundamental role. It has been reported that the presence of the inert anions influences the reaction rate due to a competition at the micellar interface with the reactive oximate.^53,57^ As a consequence different rate enhancement will take place depending on the extent to which the inert anions are bound to the micellar surface.^50,58^ The observed reaction rates are therefore in accordance with the physico-chemical data of the respective ionic liquid, as the chloride anion is less bound to the micellar surface and hence more readily exchangeable compared to bromide or iodide as it can also be seen from the *β* value calculated from the conductivity data.

This trend also exists when kinetic studies were extended to all surface-active ionic liquids involved in this study. Table 3 reports the maximum rate constants for all ionic liquids [C_12_mim]X **1–6** at a concentration close to their respective CMC, as this should correspond to the highest rate constants.

The results reported in Table 2 show again the dependence between the CMC and the rate enhancement, as ionic liquids with lower CMC show poorer performance than those with higher CMC, due to the ability of the anion to exchange with the nucleophile. Moreover, the correlation between the degree of anion binding to the micelle and the reaction rate constants exists also in the sulfonate-based series with [C_12_mim]X **4–6**. The highest rate among these surface-active ionic liquids was observed for [C_12_mim]OMs, indicating again that the careful choice of the surface-active ionic liquid can considerably affect the outcome of reactions. In fact, this suggests that it would be possible to govern the rate of a desired reaction by applying a definite micellar system with targeted surface-active ionic liquids, and various projects in this regard are currently investigated in our lab.

**Table 3:** Comparison of pseudo first order reaction rate constant of the nucleophilic substitution of nitrophenyl diphenyl phosphate (PNPDPP) with acetaldoxime at the CMC of different surface-active ionic liquids

Ionic liquid	Concentration (mM)	Rate constant 10^3^ *k*_obs_ (s^–1^)
[C_12_mim]Cl **1**	15	2.74 ± 0.46
[C_12_mim]Br **2**	10	1.30 ± 0.23
[C_12_mim]I **3**	5	0.51 ± 0.08
[C_12_mim]OMs **4**	15	1.68 ± 0.29
[C_12_mim]OTs **5**	3	0.42 ± 0.07
[C_12_mim]OTf **6**	2.5	0.18 ± 0.03
Water	—	0.08 ± 0.01

## Experimental

### Materials and methods

Commercially available reagents and solvents were used as received from Sigma Aldrich unless otherwise specified. Doubly-distilled deionized water was obtained from a Millipore Milli-Q water purification system (Millipore, USA). ^1^H and ^13^C NMR spectra were recorded on a Bruker AC 200 at 200 MHz, using the solvent peak as reference. *N*-Dodecylimidazole was synthesized according to a procedure already reported in the literature and distilled before use.^59^ All ionic liquids were dried for at least 48 h at room temperature and 0.01 mbar before use and were stored under an argon atmosphere.

### Synthetic procedures

#### Synthesis of [C_12_mim]X, X = Cl^–^, Br^–^, I^–^

The halide-based imidazolium salts were synthesized according to standard methodologies,^60^ which include the alkylation of *N*-methylimidazole with the appropriate alkyl halide to afford the corresponding imidazolium halide. The ILs were repeatedly crystallized in order to obtain colorless solids.

#### 1-Dodecyl-3-methylimidazolium chloride (**1**)

After following the general procedure the product was recrystallized from THF and obtained in 92% yield. ^1^H NMR (200 Hz, CDCl_3_): *δ* (ppm) = 0.81 (3H, t, *J* = 6.36 Hz, –C_11_H_22_–C*H*_3_), 1.18 (18H, m, –C_2_H_4_– C_9_ *H*_18_–CH_3_), 1.84 (2H, t, *J* = 6.26 Hz, –CH_2_– C*H*_2_–C_10_H_21_), 4.06 (3H, s, N–C*H*_3_), 4.25 (2H, t, *J* = 7.35 Hz, –C*H*_2_–C_11_H_23_), 7.29 (1H, s, –N–C*H*–CH–), 7.45 (1H, s, –N–C*H*–CH–), 10.54 (1H, s, –N–C*H*–N–). The data were in accordance with the literature.^61^

#### 1-Dodecyl-3-methylimidazolium bromide (**2**)

After following the general procedure the product was recrystallized from THF and obtained in 92% yield. ^1^H NMR (200 Hz, CDCl_3_): *δ* (ppm) = 0.81 (3H, t, *J* = 7.41 Hz, –C_11_H_22_–C*H*_3_), 1.18 (18H, m, –C_2_H_4_– C_9_ *H*_18_–CH_3_), 1.85 (2H, t, *J* = 6.95 Hz, –CH_2_– C*H*_2_–C_10_H_21_), 4.07 (3H, s, N–C*H*_3_), 4.25 (2H, t, *J* = 7.41 Hz, –C*H*_2_–C_11_H_23_), 7.31 (1H, s, –N–C*H*–CH–), 7.46 (1H, s, –N–C*H*–CH–), 10.35 (1H, s, –N–C*H*–N–). The data were in accordance with the literature.^5^

#### 1-Dodecyl-3-methylimidazolium iodide (**3**)

After following the general procedure the product was crystallized from toluene and further washed with *n*-hexane and obtained in 95% yield. Mp: 39 °C. C_16_H_31_IN_2_ (378.34): calcd C 50.79, H 8.26, N 7.40%; calcd (including 1.2 × H_2_O) C 48.05, H 8.42, N 7.00% found C 48.08, H 8.06, N 6.90. *ν*_max_ (cm^–1^): 3415 (O–H), 2916 (C–H), 1565(C=C), 1163 (C–N). ^1^H NMR (200 Hz, CDCl_3_): *δ* (ppm) = 0.82 (3H, m, –C_11_H_22_–C*H*_3_), 1.19 (18H, m, –C_2_H_4_– C_9_ *H*_18_–CH_3_), 1.88 (2H, m, –CH_2_– C*H*_2_–C_10_H_21_), 4.08 (3H, s, N–C*H*_3_), 4.27 (2H, t, *J* = 7.36 Hz, –C*H*_2_–C_11_H_23_), 7.45 (1H, s, –N–C*H*–CH–), 7.59 (1H, s, –N–C*H*–CH–), 9.94 (1H, s, –N–C*H*–N–). ^13^C NMR (200 Hz, CDCl_3_): *δ* (ppm) = 136.59, 123.84, 122.18, 50.20, 37.07, 31.82, 30.23, 29.52 (2C), 29.44, 29.32, 29.25, 28.93, 26.18, 22.60, 14.06.

#### 1-Dodecyl-3-methylimidazolium methanesulfonate (**4**)

*N*-Dodecylimidazole (27.06 g, 30 mmol) was dissolved in 8 ml ethyl acetate, and a solution of methyl methanesulfonate (3.47 g, 31.5 mmol) in 8 ml ethyl acetate was added dropwise at RT. The reaction mixture was then stirred for 6 h at 50 °C. The product precipitated upon cooling and was recrystallized two more times to obtain the product in high purity. After removal of solvent traces under reduced pressure for at least 2 d at 0.01 mbar, 1-dodecyl-3-methylimidazolium methanesulfonate was obtained as a colorless powder in 83% yield. ^1^H NMR (200 Hz, CDCl_3_): *δ* (ppm) = 0.88 (3H, t, *J* = 6.17 Hz, –C_11_H_22_–C*H*_3_), 1.25 (18H, m, –C_2_H_4_– C_9_ *H*_18_–CH_3_), 1.88 (2H, m, –CH_2_– C*H*_2_–C_10_H_21_), 2.83 (3H, s, –S–C*H*_3_), 4.06 (3H, s, N–C*H*_3_), 4.26 (2H, t, *J* = 7.33 Hz, –C*H*_2_–C_11_H_23_), 7.21 (1H, s, –N–C*H*–CH–), 7.27 (1H, s, –N–C*H*–CH–), 10.10 (1H, s, –N–C*H*–N–). The data were in accordance with the literature.^32^

#### 1-Dodecyl-3-methylimidazolium *p*-toluenesulfonate (**5**)

*N*-Dodecylimidazole (2.85 g, 12 mmol) was dissolved in 8 ml ethyl acetate, and a solution of methyl *p*-toluenesulfonate (2.37 g, 12.6 mmol) in 8 ml ethyl acetate was added dropwise at RT. The reaction mixture was then stirred for 3 h at 50 °C. The product precipitated upon cooling and was recrystallized two more times to obtain the product in high purity. After removal of solvent traces under reduced pressure for at least 2 d at 0.01 mbar, 1-dodecyl-3-methylimidazolium *p*-toluenesulfonate was obtained as a colorless crystal in 85% yield. Mp: 97 °C. C_23_H_38_N_2_O_3_S (422.63): calcd C 65.37, H 9.06, N 6.63%; found C 65.42, H 9.20, N 6.66. *ν*_max_ (cm^–1^): 2919 (C–H), 1575 (C=C), 1498 (S=O), 1194 (Ar–C–C), 1034 (C–N). ^1^H NMR (200 Hz, CDCl_3_): *δ* (ppm) = 0.81 (3H, t, *J* = 12.8 Hz, –C_11_H_22_–C*H*_3_), 1.16 (18H, m, –C_2_H_4_– C_9_ *H*_18_–CH_3_), 1.71 (2H, t, *J* = 6.55 Hz, –CH_2_– C*H*_2_–C_10_H_21_), 2.27 (3H, s, –C_6_H_6_–C*H*_3_), 3.91 (3H, s, N–C*H*_3_), 4.09 (2H, t, *J* = 14.87 Hz, –C*H*_2_–C_11_H_23_), 7.07 (2H, d, *J* = 7.98 Hz, aromatic proton), 7.18 (1H, s, –N–C*H*–CH–), 7.32 (1H, s, –N–C*H*–CH–), 7.71 (2H, d, *J* = 8.12 Hz, aromatic proton), 9.78 (1H, s, –N–C*H*–N–). ^13^C NMR (50 Hz, CDCl_3_): *δ* (ppm) = 143.91, 139.25, 137.6, 137.44, 128.60, 125.73, 123.79, 122.01, 49.74, 36.15, 31.83, 30.87, 30.14, 29.55–29.48 (3C), 29.34–29.27 (2C), 28.93, 26.13, 22.61, 21.21, 14.06.

#### 1-Dodecyl-3-methylimidazolium trifluoromethane sulfonate (**6**)

*N*-Dodecylimidazole (5.16 g, 21.8 mmol) was dissolved in 25 ml anhydrous DCM in argon and freshly distilled methyl trifluoromethane sulfonate (4.3 g, 3 ml, 26.2 mmol) was added dropwise while cooling in an ice bath. The reaction mixture was then stirred for 3 h at RT until NMR showed complete conversion. The volatile materials were removed under reduced pressure, and the product was repeatedly washed with *n*-hexane. 1-Dodecyl-3-methylimidazolium trifluoromethane sulfonate was obtained *via* filtration as a pale solid in 97% yield. ^1^NMR (CDCl_3_): *δ* (ppm) = 0.87 (3H, t, *J* = 6.36 Hz, –C_11_H_22_–C*H*_3_), 1.25 (18H, m, –C_2_H_4_– C_9_ *H*_18_–CH_3_), 1.87 (2H, br, –CH_2_–C*H*_2_–C_10_H_21_), 3.98 (3H, s, N–C*H*_3_), 4.18 (2H, t, *J* = 7.24 Hz, –C*H*_2_–C_11_H_23_), 7.31 (1H, s, –N–C*H*–CH–), 7.38 (1H, s, –N–C*H*–CH–), 9.13 (1H, s, –N–C*H*–N–). The data were in accordance with the literature.^62^

### Characterization of surface-activity

Solutions at various concentrations of the ionic liquids were prepared with doubly-distilled Millipore Milli-Q water and left under shaking at 360 min^–1^ for 24 h at RT to equilibrate.

#### Conductivity measurements

The samples were previously equilibrated at (25 ± 0.1) °C in a HAAKE K15 thermostat, then conductivity measurements were performed on a Mettler Toledo Seven Excellence system, equipped with an InLAB® 741-ISM electrode (cell constant *k* = 0.105). The conductometer was calibrated with a standard KCl solution and two independent measurements were performed. The CMC was calculated as the intersection point of the two linear regimes in the conductivity/concentration graph. The degree of counter ion binding was calculated using the following equation *β* = 1 – *α*, where *α* is the degree of ionization and corresponds to the ratio of the slopes between the two linear fragments of the conductivity curves.

#### Surface tension measurements

Surface tension was determined using the Du Noüy ring method on a Krüss manual tensiometer K6 at RT. Each measurement was repeated 5 times.

#### UV-Vis measurements

For the UV-Vis measurements a stock solution of benzoylacetone (31 mM) in 1,4-dioxane was prepared, which was used to prepare a diluted solution in water (0.5 mM). For UV-Vis measurements 0.2 ml of benzoylacetone solution in water were added to the ionic liquid solution to obtain a final volume of 1.5 ml. These solutions were equilibrated at 25 °C for at least 1 h, and then transferred to the cuvette for measurement. The final concentration of benzoylacetone in the cuvette was 0.07 mM, while the reference cuvette contained all reagents except for benzoylacetone.

#### Small angle X-ray scattering

Small-angle scattering experiments were performed using a Bruker Nanostar (Bruker AXS), equipped with a microfocus X-ray source (Incoatec IμS High Brilliance) with CuKα radiation with a wavelength of 0.1542 nm and a 2D-position sensitive detector (Vantec 2000). The scattering patterns were recorded for 2 hours. The data were azimuthally integrated and corrected for background scattering resulting in scattering intensities *I*(*q*) depending on the scattering vector *q* = (4π/*λ*)sin *θ* in the range from *q* = 0.1 to 20 nm^–1^, with 2*θ* being the scattering angle. In the figures, only the range up to *q* = 5 nm^–1^ is shown for clarity.

#### Micellar catalysis

4-Nitrophenyl diphenyl phosphate (PNPDPP) was synthesized according to an already reported procedure^63^ and crystallized from ethanol. Due to its low solubility in water a stock solution in acetonitrile was prepared (11.5 × 10^–3^ M). In parallel, a stock buffer solution of acetaldehydeoxime (552 × 10^–3^ M) in water was prepared by using a NaOH solution as solvent in order to obtain a NaOH : oxime ratio of 1 : 2.

#### Kinetic measurements

Kinetics experiments were performed by UV-Visible spectroscopy using a Shimadzu UV1800 spectrometer equipped with a thermostate at 25 °C. All kinetic runs were performed under pseudo first order conditions in which the concentration of the oxime was at least 100 times greater than the initial concentration [PNPDPP]_0_. Reaction mixtures were always freshly prepared by first mixing the 2.4 ml ionic liquid solution with 10 μl oxime stock solutions. The kinetic run was then started by adding 2 μl of the PNPDPP stock solution to 1 ml of ionic liquid–oxime solution in a quartz cuvette. The initial concentrations of PNPDPP and oxime in the cuvette were 2.3 × 10^–5^ M and 2.3 × 10^–3^ M respectively. The formation of the product *p*-nitrophenolate NP was monitored at a fixed wavelength of 402.5 nm. Generally each reaction was followed until no change in the final absorbance was observed anymore. The rate constants for both the desired reaction, and the by-product formation, were calculated through least-squares exponential fitting, the system was assimilated in the case of parallel reactions. The following formula afforded the rate constants for the formation of product *p*-nitrophenolate NP:
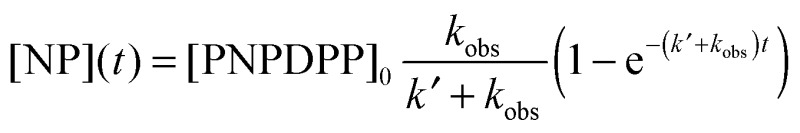
The reported values are an average of at least 3 runs. The values of the rate constant of the parallel reaction *k*′ were always at least one order of magnitude smaller than *k*_obs_.

#### Theoretical final absorbance

The theoretical final absorbance values for the reaction between PNPDPP and the oxime were measured by injecting 2 μl of an acetonitrile solution of *p*-nitrophenol (11.5 × 10^–3^ M, equivalent to the PNPDPP in the kinetic study) in 1 ml of the corresponding IL–oxime solution. Each value was taken two times.

#### ^31^P NMR measurements

Samples in DMSO–water [10 : 90 (w/w)] were measured containing either the starting material or the reaction mixture. The peaks were referred to a capillary with H_3_PO_4_ as external standard. The obtained chemical shifts are in good agreement with those reported in the literature, and no other products were identified.

## Conclusions

We have reported a study on the counterion role in surface-active ionic liquids based on 1-dodecyl-3-methylimidazolium. The two investigated series based on halides and sulfonate anions show a correlation between the tendency of the anions to reside closer to the micellar interface and their surface activity. These properties were reflected also when the surface-active ionic liquids were implied in micellar catalysis, in fact, a different rate acceleration was obtained using the different ionic liquids in nucleophilic substitutions in micellar media.
