# Supplementary material for: Surface-active ionic liquids in micellar catalysis: impact of anion selection on reaction rates in nucleophilic substitutions
Source: Phys Chem Chem Phys. 2016 Apr 28;18(19):13375–84. doi: 10.1039/c6cp00493h (PMC5317217; doi:10.1039/c6cp00493h)
Supplement: Supplementary file 1 [file CP-018-C6CP00493H-s001.pdf]

Electronic Supplementary Material (ESI) for Physical Chemistry Chemical Physics.
This journal is © the Owner Societies 2016

Supporting informations

Surface-active ionic liquids in micellar catalysis: Impact of anion selection on reaction rates in nucleophilic substitutions

Alice Cognigni,[a] Peter Gaertner,[a] Ronald Zirbs,[b] Herwig Peterlik,[c] Katharina Prochazka,[c] Christian Schröder,[d] and Katharina Bica*,[a]

[a]Institute of Applied Synthetic Chemistry, Vienna University of Technology, Getreidemarkt 9/163, 1060 Vienna, Austria.

[b]Group for Biologically Inspired Materials, Institute of Nanobiotechnology (DNBT), University of Natural Resources and Life Sciences, Muthgasse 11, 1190 Vienna, Austria.

[c]Dynamics of Condensed Systems, University of Vienna, Boltzmanngasse 5, 1090 Vienna, Austria.

[d]Institute of Computational Biological Chemistry, University of Vienna, Währingerstrasse 17, 1090 Vienna, Austria

*Corresponding author Katharina Schröder (née Katharina Bica). Email: katharina.schroeder@tuwien.ac.at

Table of contents

1. Thermodynamic properties calculation 2

2. Kinetic constants fitting 3

3. Additional SAXS data 5

4. ^{1}H and ^{13}C NMR spectra of the surface active ILs 7

1. CMC determination and aggregation parameters calculation

For all the techniques implied the CMC was determined at the break point, i.e. the intersection of the two linear fittings of data points.

Surface tension

Π_{CMC} is the effectiveness of the surface tension reduction, it is defined according to the following equation[1] (1):

$$\Pi_{CMC} = \gamma_0 - \gamma_{CMC,} \tag{1}$$

where γ_0 is the surface tension of water, and γ_{CMC} is the surface tension of the solution at the CMC.

The Gibbs adsorption isotherm equation can be used to estimate the average area per molecule residing at the surface (A_{min}), assuming that the structure pattern corresponds to a monolayer, it is defined according to the following equations[2] (2-3):

$$A_{min}=10^{21}/N\Gamma \ [nm^2] \tag{2}$$

where N= Avogadro's number, and Γ=surface excess concentration [mol/1000 m^2]

$$\Gamma = - \frac{1}{4.606\ RT}\left(\frac{d\gamma}{d\ log\ C_1}\right)_T \tag{3}$$

where $\frac{d\gamma}{d\ log\ C_1}$ is the slope of the curve surface tension vs logC before the CMC, and the concentration is expressed in mol/L, and R=8.31 J /mol K.

The packing parameter[3] (P) which includes the volume (v) of the hydrocarbon chain imbedded in the hydrocarbon core of the aggregate and the maximum effective length (l_c) that the chain can assume, calculated according to Tanford equation (6-7)[37]:

$$v= (27.4+26.9 * n)*10^{-3}\ nm^3 \tag{6}$$
$$l_c= (0.154 + 0.1265 * n)\ nm \tag{7}$$

n equals the actual number of atom in the hydrophobic core of the micelle, so generally one is left out.

The packing parameter is then defined as $P=v/a_0l_c$, where a_0 is the cross-sectional area occupied by the hydrophilic group at the micelle-solution interface, it is in our case approximated with A_{min}. A value of $P < 0.33$ corresponds to spherical micelles, and intermediate value $0.33<P<0.5$ corresponds

[1]Blesic, M.; Swadźba-Kwaśny, M.; Holbrey, J. D.; Canongia Lopes, J. N.; Seddon, K. R.; Rebelo, L. P. N. *Physical Chemistry Chemical Physics* 2009, **11**, 4260.
[2] Milton J. Rosen and Joy T. Kunjappu, *Surfactants and interfacial phenomena*, John Wiley & Sons, 4th end., 2012.
[3] J. N. Israelachvili, in *Intermolecular and Surface Forces*, Elsevier, 2011, pp. 535–576.

to non-spherical, ellipsoidal micelles, while higher values correspond to rod-like micelles and various interconnected structures.[36]

Conductivity

The degree of counterion binding (β) can be estimated from the conductivity data, based on the ratio of the slopes above (S_2) and below (S_1) the cmc[4] (4):

$$\beta = (1 - S_2/S_1) \qquad (4)$$

The Gibbs energy of micellization (ΔG^0_{mic}) was calculated form the conductivity data according to the following equation[5] (5):

$$\Delta G^0_{mic} = (1+ \beta)\ RT \ln (CMC/W) \qquad (5)$$

Where the argument of the logarithms is the molar fraction of the surfactant, and water concentration is taken as W=55.4 mol/L.

[4] Ao, M.; Kim, D. *Journal of Chemical & Engineering Data* 2013, **58**, 1529.
[5] R. Zana, *Langmuir*, 1996, **12**, 1208.

2. Kinetic constants fitting

The investigated reaction between 4-nitrophenyl diphenyl phosphate (**PNPDPP**) and acetaldoxime follows a follows a $S_N2(P)$ mechanism via in the attack of the nucleophile acetaldoxime at the phosphorous centre. Two leaving groups are possible, either the favoured *p*-nitrophenolate (**NP**), whose formation can be followed at 402.5 nm or phenolate (**P**) as a by-product.

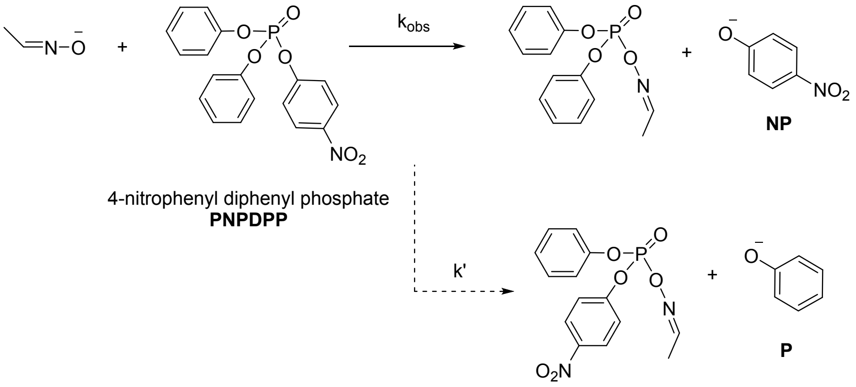

For the determination of the rate constants for both, the desired reaction (k_{obs}) and the by-product formation (k'), a competitive reaction scheme (see figure above) is assumed which leads to a system of coupled differential equations:

$$\frac{d\vec{c}(t)}{dt} = \boldsymbol{R} \cdot \vec{c}(t) \qquad (1)$$

where the vector $\vec{c}$(t) represents the concentration of PNPDPP, NP and P at time t

$$\vec{c}(t) = \begin{pmatrix} [\mathrm{PNPDPP}](t) \\ [\mathrm{NP}](t) \\ [\mathrm{P}](t) \end{pmatrix} \qquad (2)$$

and the reaction matrix **R** looks like

$$\boldsymbol{R} = \begin{pmatrix} -k_{obs} - k' & 0 & 0 \\ k_{obs} & 0 & 0 \\ k' & 0 & 0 \end{pmatrix} \qquad (3)$$

The general solution of the coupled differential equations in Eq. (1) depends on the Eigenvalues λ_1=0, λ_2=0 and λ_3=-(k_{obs} +k') and the corresponding Eigenvectors

$$\vec{e}_1 = \begin{pmatrix} 0 \\ 0 \\ 1 \end{pmatrix} \qquad \vec{e}_2 = \begin{pmatrix} 0 \\ 1 \\ 0 \end{pmatrix} \qquad \vec{e}_3 = \frac{1}{k'} \begin{pmatrix} -(k_{obs} + k') \\ k_{obs} \\ k' \end{pmatrix}$$

of the reaction matrix **R**:

$$\vec{c}(t) = \sum_{i=1}^{3} \alpha_i \vec{e}_i e^{\lambda_i t} \qquad (4)$$

At time t=0, $\vec{c}(0)$ = ($[\mathrm{PNPDPP}]_0$, 0, 0) since only the reactant is present. Using this initial condition, the α-coefficients can be determined:

$$\alpha_3 = -[\mathrm{PNPDPP}]_0 \frac{k'}{k_{obs} + k'}$$

$$\alpha_2 = [\mathrm{PNPDPP}]_0 \frac{k_{obs}}{k_{obs} + k'}$$

$$\alpha_1 = [\mathrm{PNPDPP}]_0 \frac{k'}{k_{obs} + k'} = -\alpha_3$$

As a result, the time-dependent concentration of NP yields

$$[\mathrm{NP}](t) = [\mathrm{PNPDPP}]_0 \quad \frac{k_{obs}}{k_{obs} + k'} \left(1 - e^{-(k_{\mathrm{obs}} + k')t}\right) \tag{5}$$

In a least-square fit of the spectroscopic data to Eq. (5) using gnuplot, the sum of the two rate constants k_{obs} + k' can be determined. The individual values of k_{obs} and k' are evaluated from the ratio of the initial concentration of the reactant $[PNPDPP]_0$ and the final concentration of the product NP which also equals k_{obs} / (k_{obs}+k').

3. Additional SAXS data

The scaled medium contrast is obtained by the ratio of the electron densities (ref. 35 of the paper, P. Bartlett and R.H. Ottewill, *J. Chem. Phys.*, 1992, **96**, 3306)

$$\gamma = \frac{\rho_m - \rho_s}{\rho_c - \rho_s}$$

where the subscript denotes (surrounding) **m**atrix, **s**hell and **c**ore, respectively. $\gamma = 0$ means that density of core and medium are identical, $\rho_m = \rho_s$, and only the core scatters. For $\gamma = 1$, $\rho_m = \rho_c$ and only the shell contributes to scattering.

The following Figures S1a to S1c show the shift towards a more uniform distribution of the radius of the core for higher concentrations. It may be not the case for Cl (as these data are difficult to evaluate due to the weakly pronounced maximum in the scattering intensities), but clearly visible for Br and OTS.

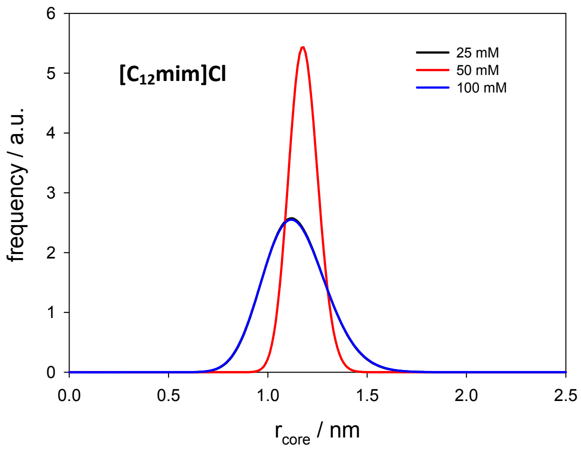

Fig. S1a: Size distribution of core radius for different concentrations of [C_{12}mim]Cl.

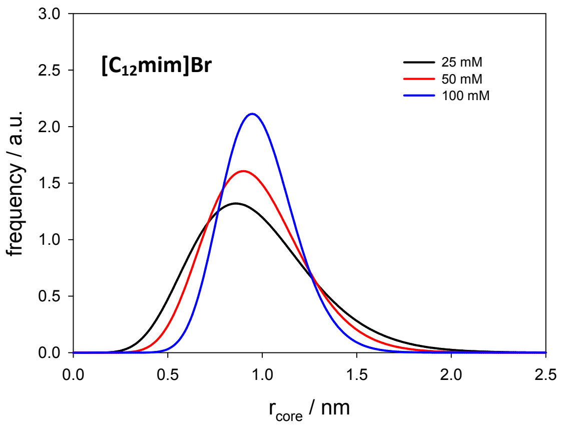

Fig. S1b: Size distribution of core radius for different concentrations of [C_{12}mim]Br.

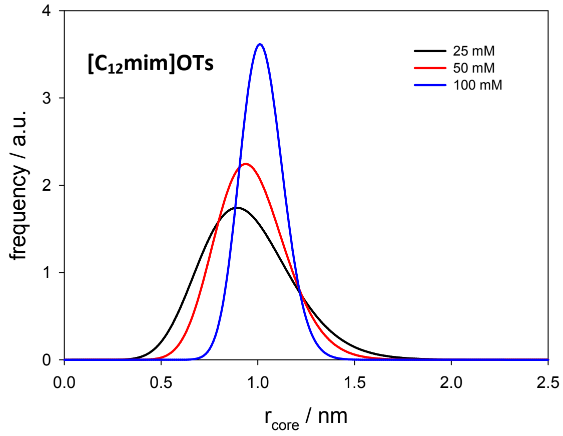

Fig. S1c: Size distribution of core radius for different concentrations of [C_{12}mim]OTs.

4. ^{1}H and ^{13}C NMR spectra of the surface active ILs

$[C_{12}mim]Cl$

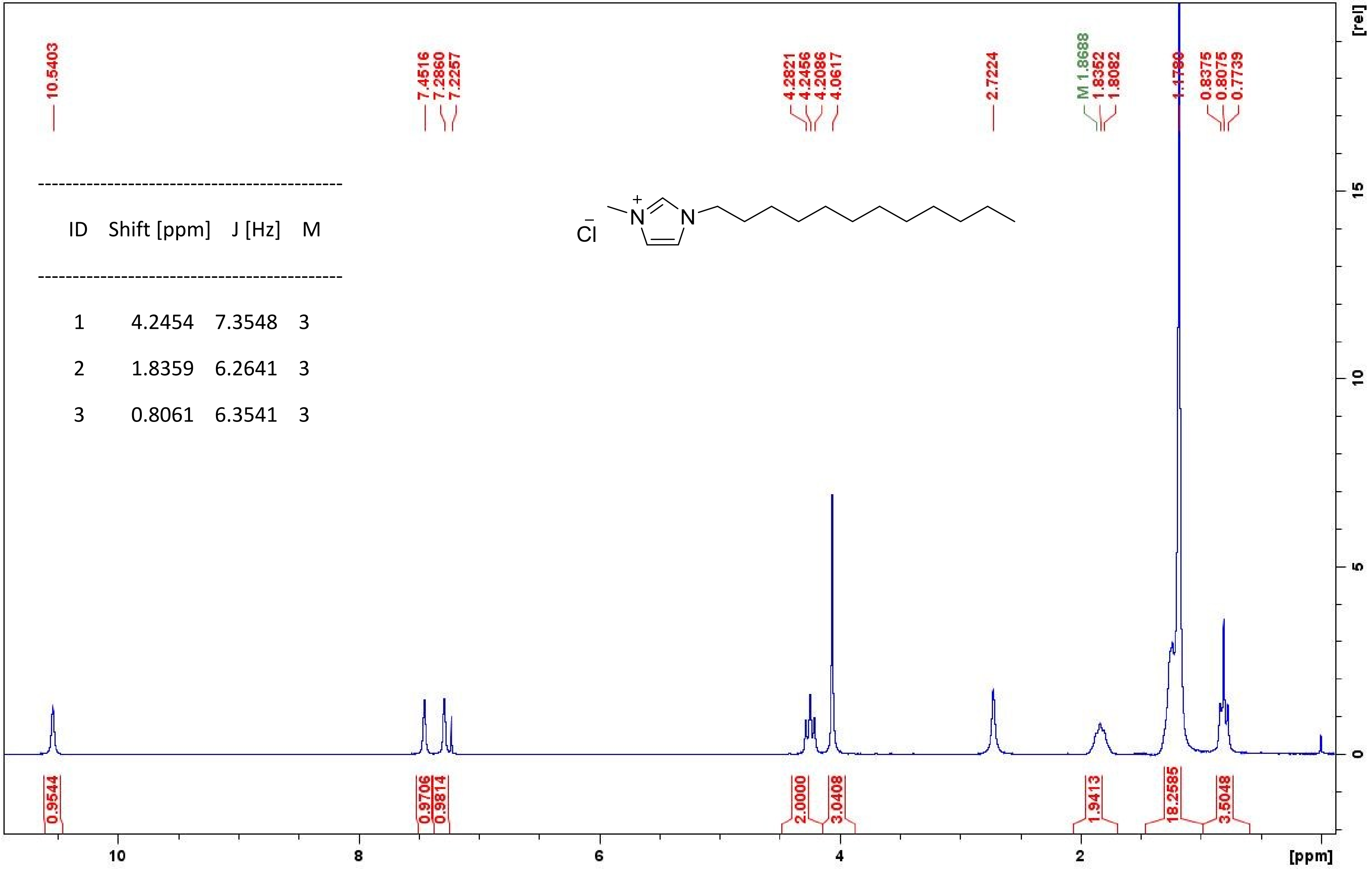

[C_{12}mim]Br

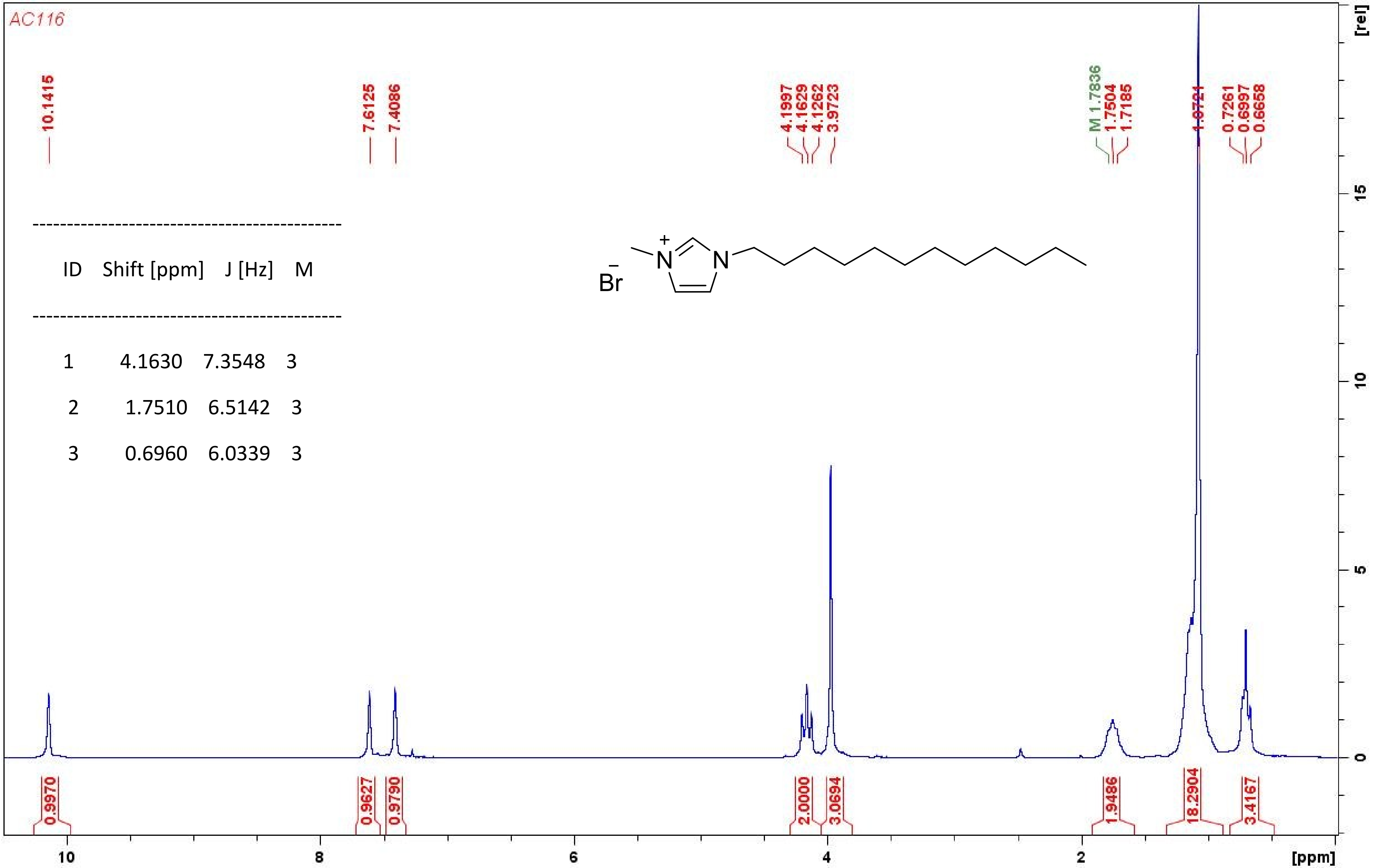
AC116
10.1415
7.6125
7.4086
4.1997
4.1629
4.1262
3.9723
M 1.7836
1.7504
1.7185
0.7261
0.6997
0.6658
ID Shift [ppm] J [Hz] M
1 4.1630 7.3548 3
2 1.7510 6.5142 3
3 0.6960 6.0339 3
Br
N
N
0.9970
0.9627
0.9790
2.0000
3.0694
1.9486
18.2904
3.4167
[rel]
[ppm]

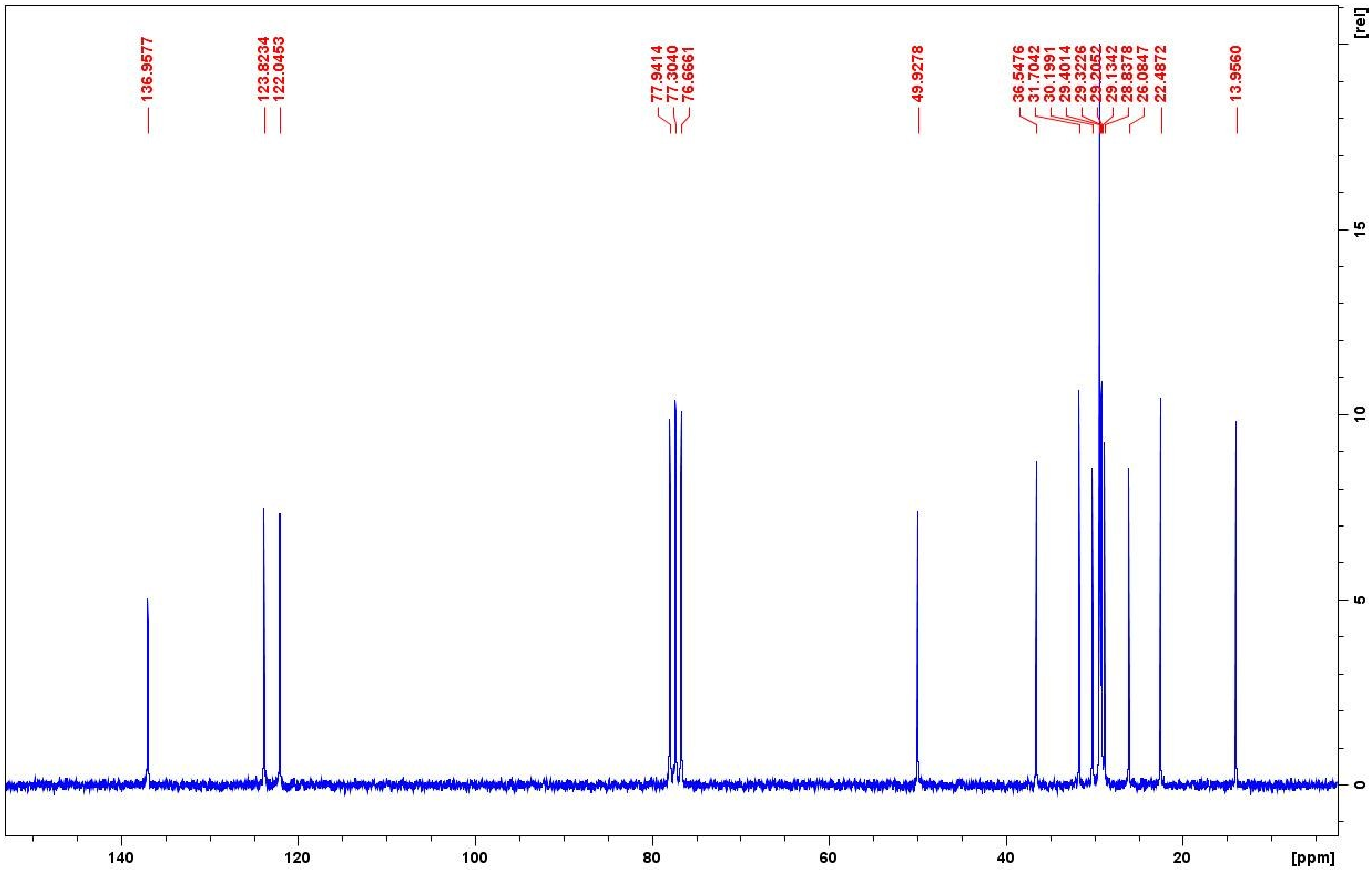
136.9577
123.8234
122.0453
77.9414
77.3040
76.6661
49.9278
36.5476
31.7042
30.1991
29.4014
29.3226
29.2052
29.1342
28.8378
26.0847
22.4872
13.9560
[rel]
15
10
5
0
140
120
100
80
60
40
20
[ppm]

[C_{12}mim]I

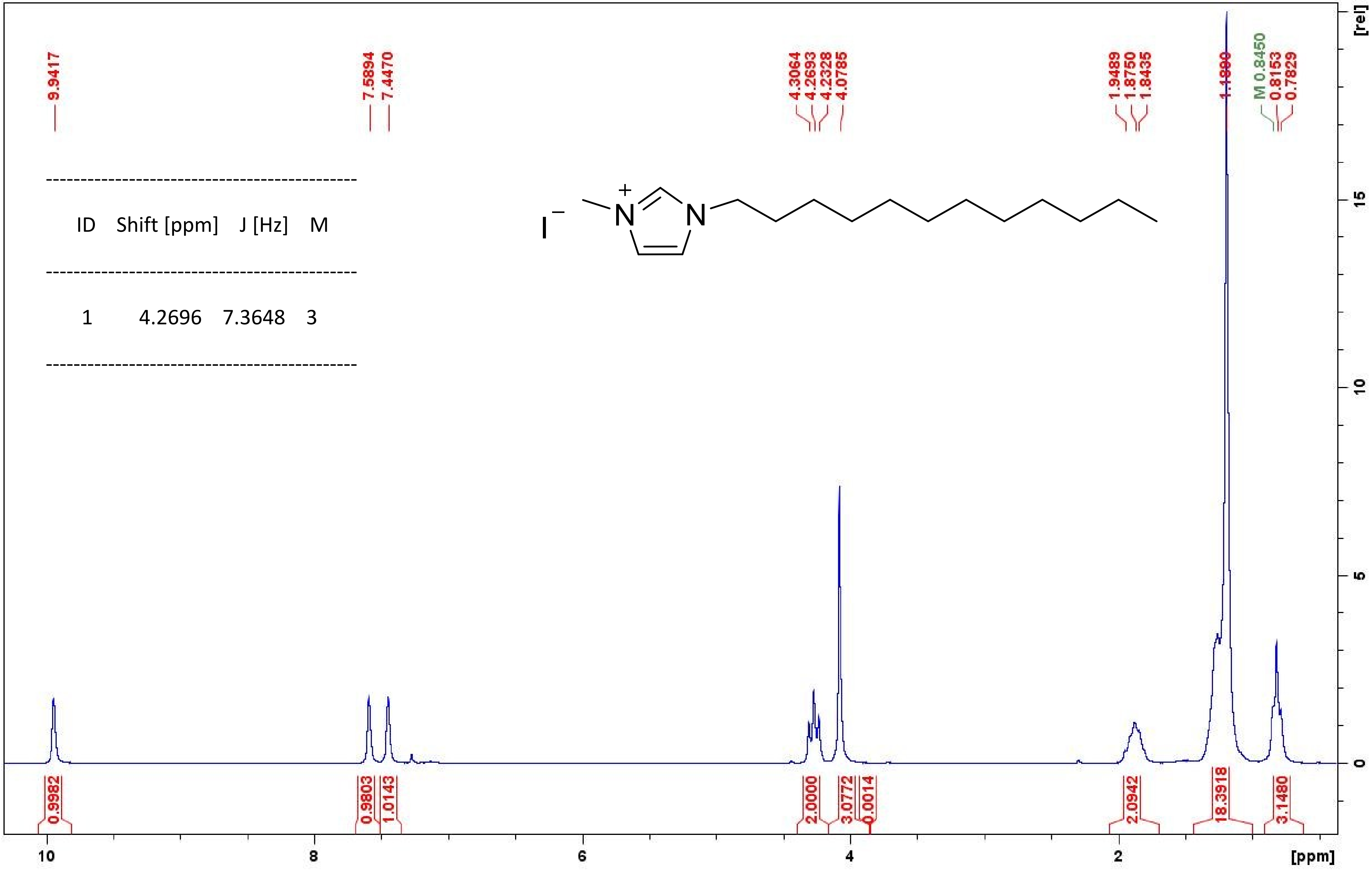

9.9417
7.5894
7.4470
4.3064
4.2693
4.2328
4.0785
1.9489
1.8750
1.8435
1.1890
M 0.8450
0.8153
0.7829
ID Shift [ppm] J [Hz] M
1 4.2696 7.3648 3
I−
N+
N
0.9982
0.9803
1.0143
2.0000
3.0772
0.0014
2.0942
18.3918
3.1480
[rel]
[ppm]

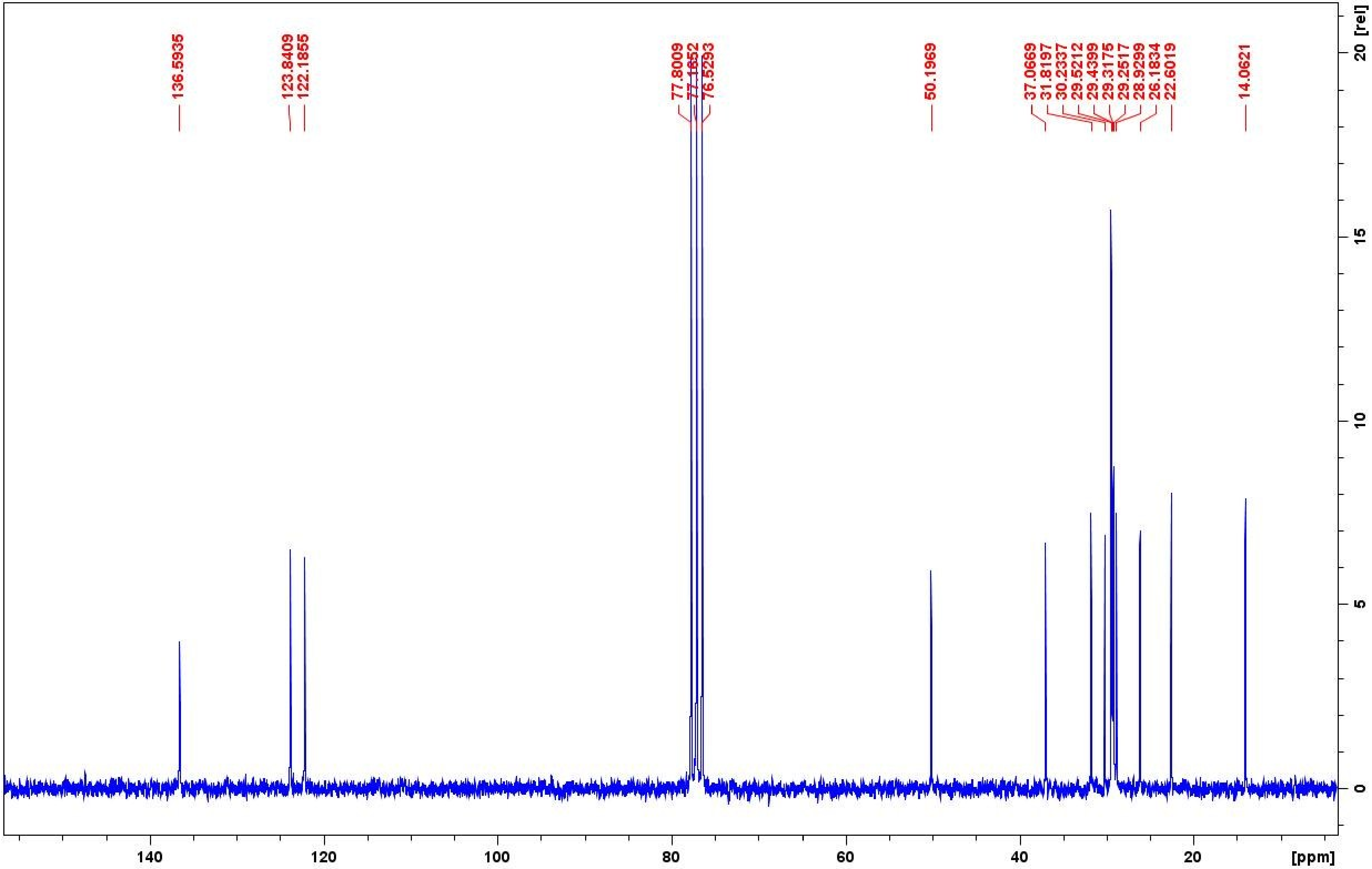
136.5935
123.8409
122.1855
77.8009
77.1652
76.5293
50.1969
37.0669
31.8197
30.2337
29.5212
29.4399
29.3175
29.2517
28.9299
26.1834
22.6019
14.0621
[rel]
20
15
10
5
0
140
120
100
80
60
40
20
[ppm]

[C_{12}mim]OMs

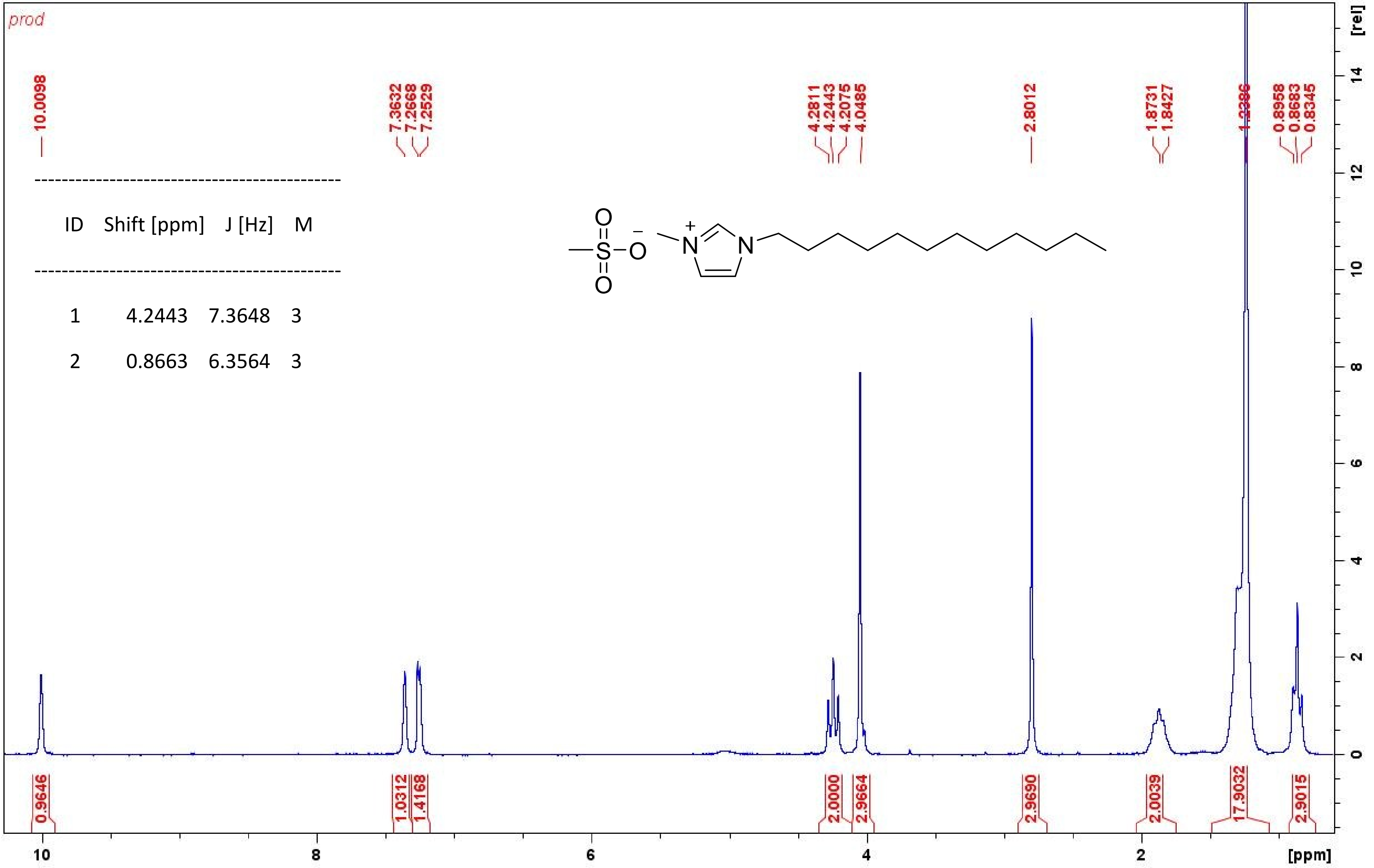
prod
10.0098
7.3632
7.2668
7.2529
4.2811
4.2443
4.2075
4.0485
2.8012
1.8731
1.8427
0.8958
0.8683
0.8345
ID Shift [ppm] J [Hz] M
1 4.2443 7.3648 3
2 0.8663 6.3564 3
0.9646
1.0312
1.4168
2.0000
2.9664
2.9690
2.0039
17.9032
2.9015
10
8
6
4
2
[ppm]
[rel]
0
2
4
6
8
10
12
14

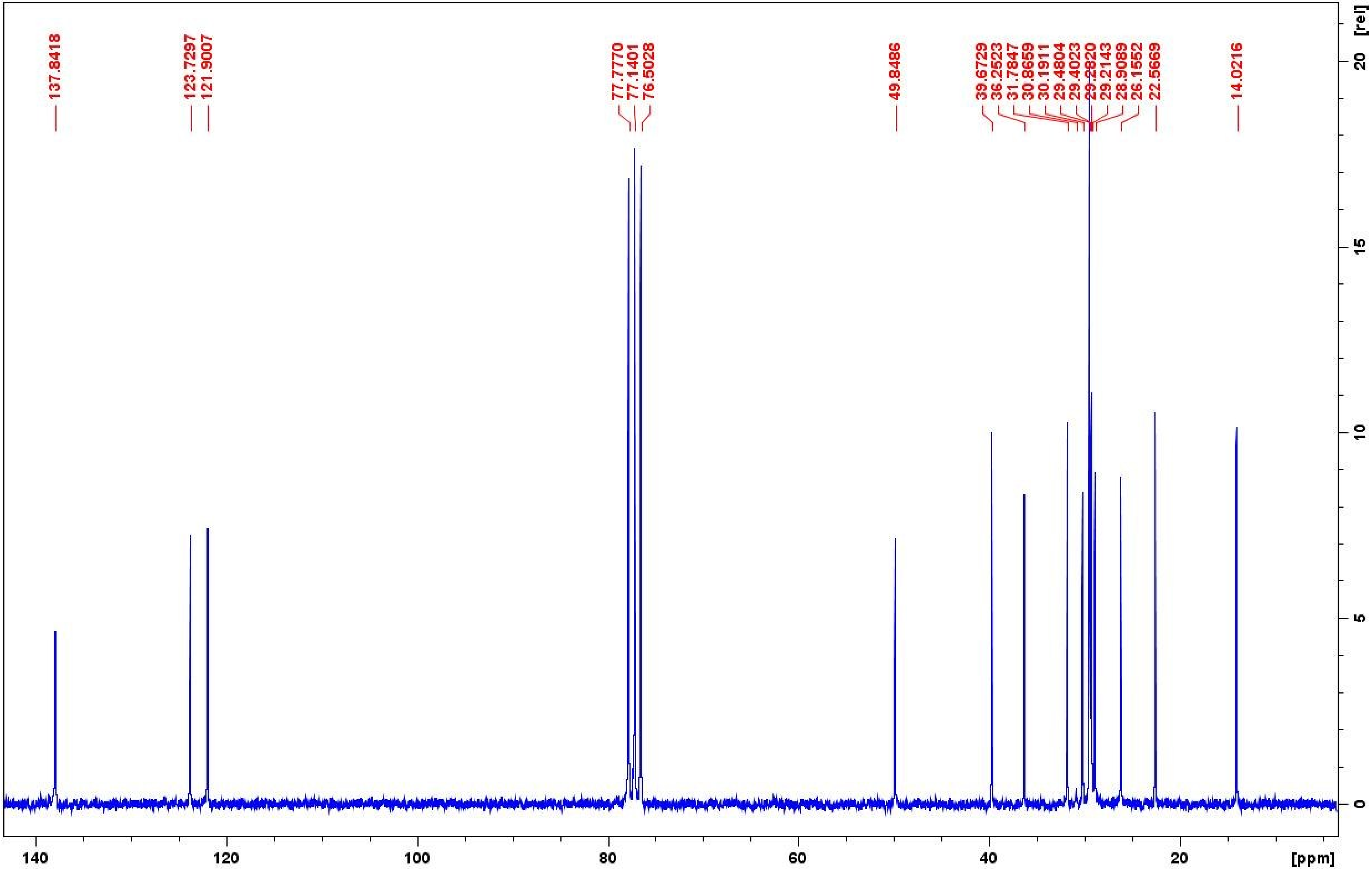
137.8418
123.7297
121.9007
77.7770
77.1401
76.5028
49.8486
39.6729
36.2523
31.7847
30.8659
30.1911
29.4804
29.4023
29.2820
29.2143
28.9089
26.1552
22.5669
14.0216
[rel]
[ppm]

[C_{12}mim]OTs

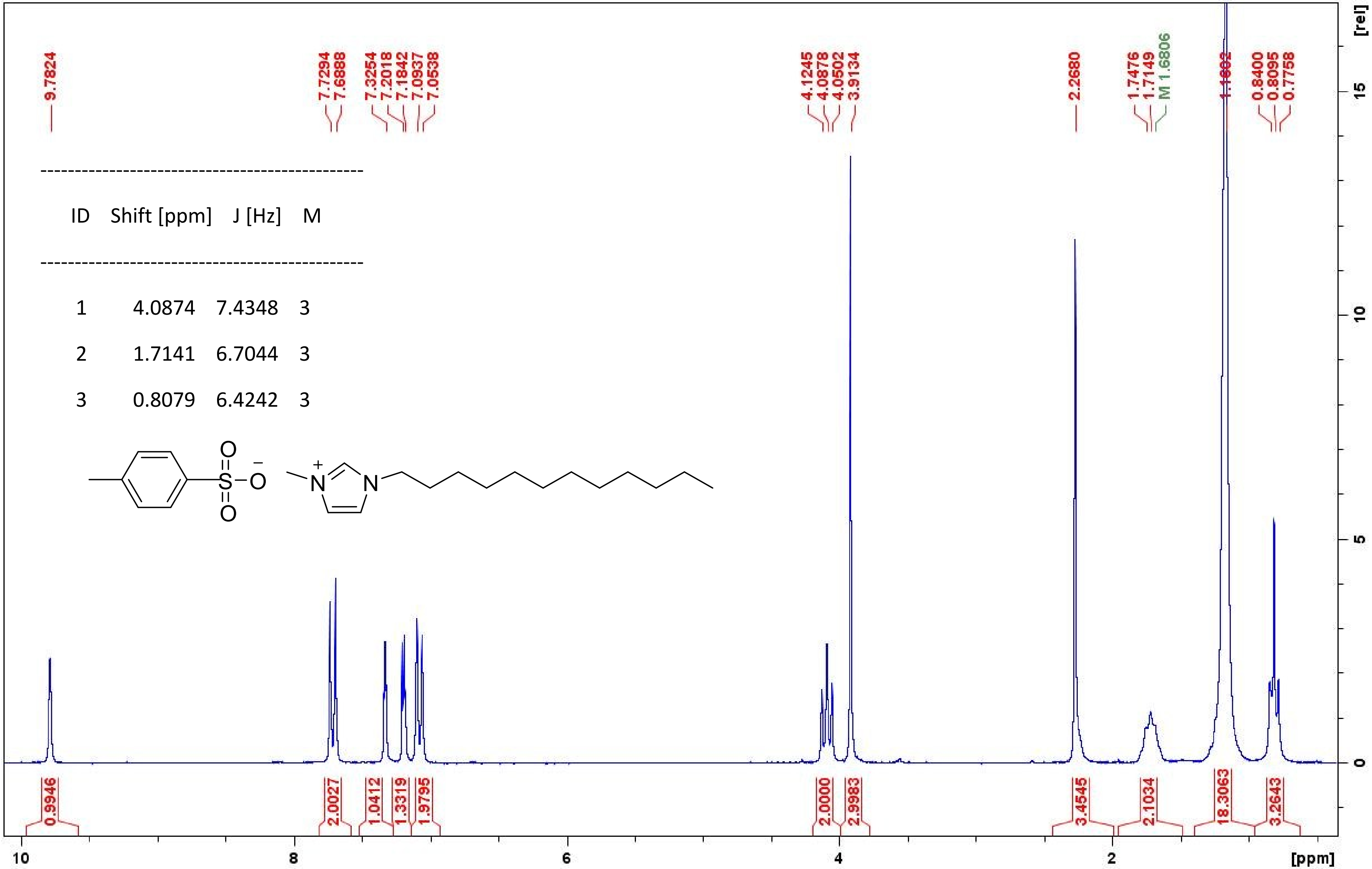
9.7824
7.7294
7.6888
7.3254
7.2018
7.1842
7.0937
7.0538
4.1245
4.0878
4.0502
3.9134
2.2680
1.7476
1.7149
M 1.6806
0.8400
0.8095
0.7758
ID Shift [ppm] J [Hz] M
1 4.0874 7.4348 3
2 1.7141 6.7044 3
3 0.8079 6.4242 3
O
S
O
O
N
N
0.9946
2.0027
1.0412
1.3319
1.9795
2.0000
2.9983
3.4545
2.1034
18.3063
3.2643
10
8
6
4
2
[ppm]
[rel]
15
10
5
0

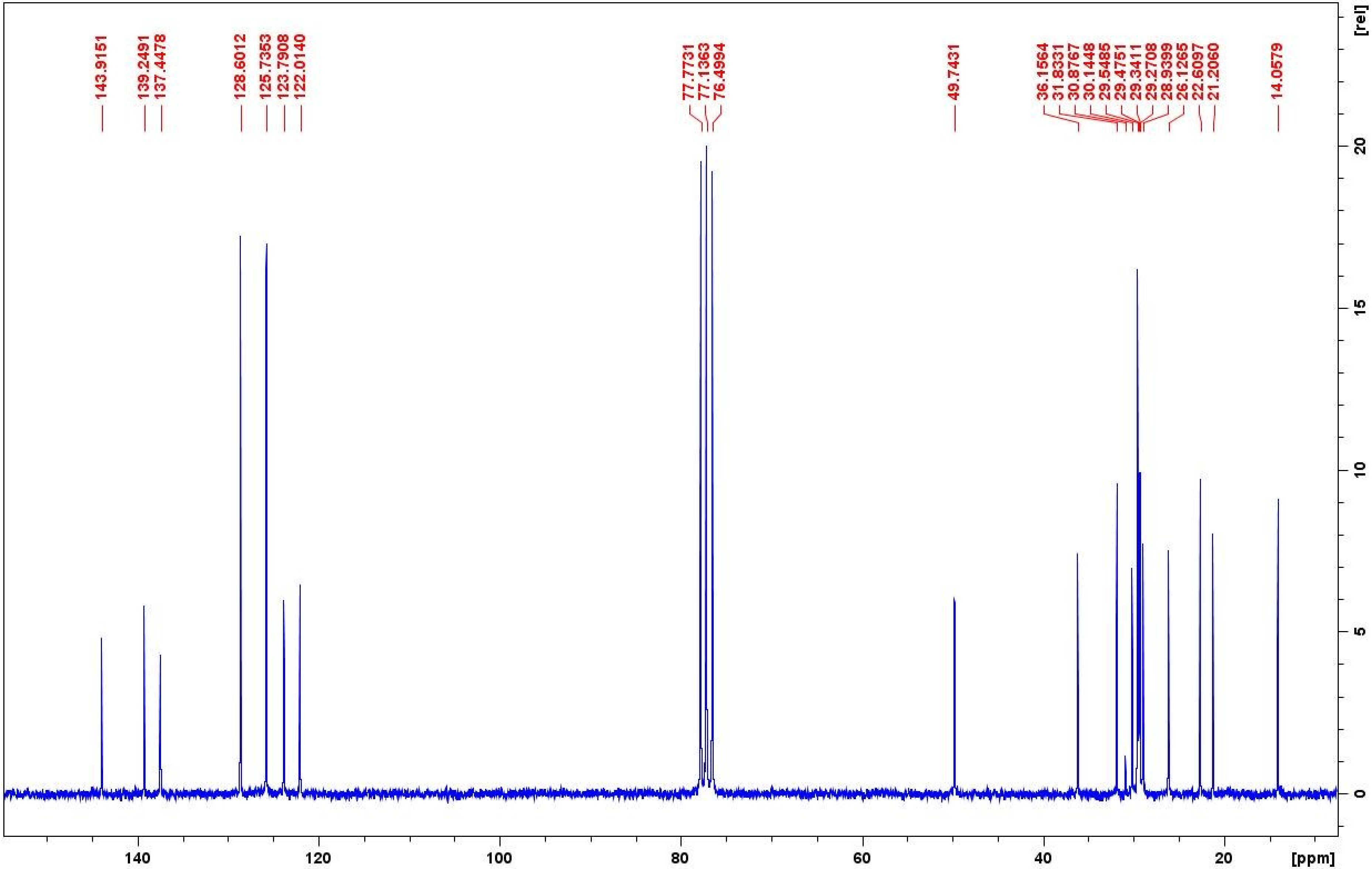
143.9151
139.2491
137.4478
128.6012
125.7353
123.7908
122.0140
77.7731
77.1363
76.4994
49.7431
36.1564
31.8331
30.8767
30.1448
29.5485
29.4751
29.3411
29.2708
28.9399
26.1265
22.6097
21.2060
14.0579
[rel]
20
15
10
5
0
140
120
100
80
60
40
20
[ppm]

[C_{12}mim]OTf

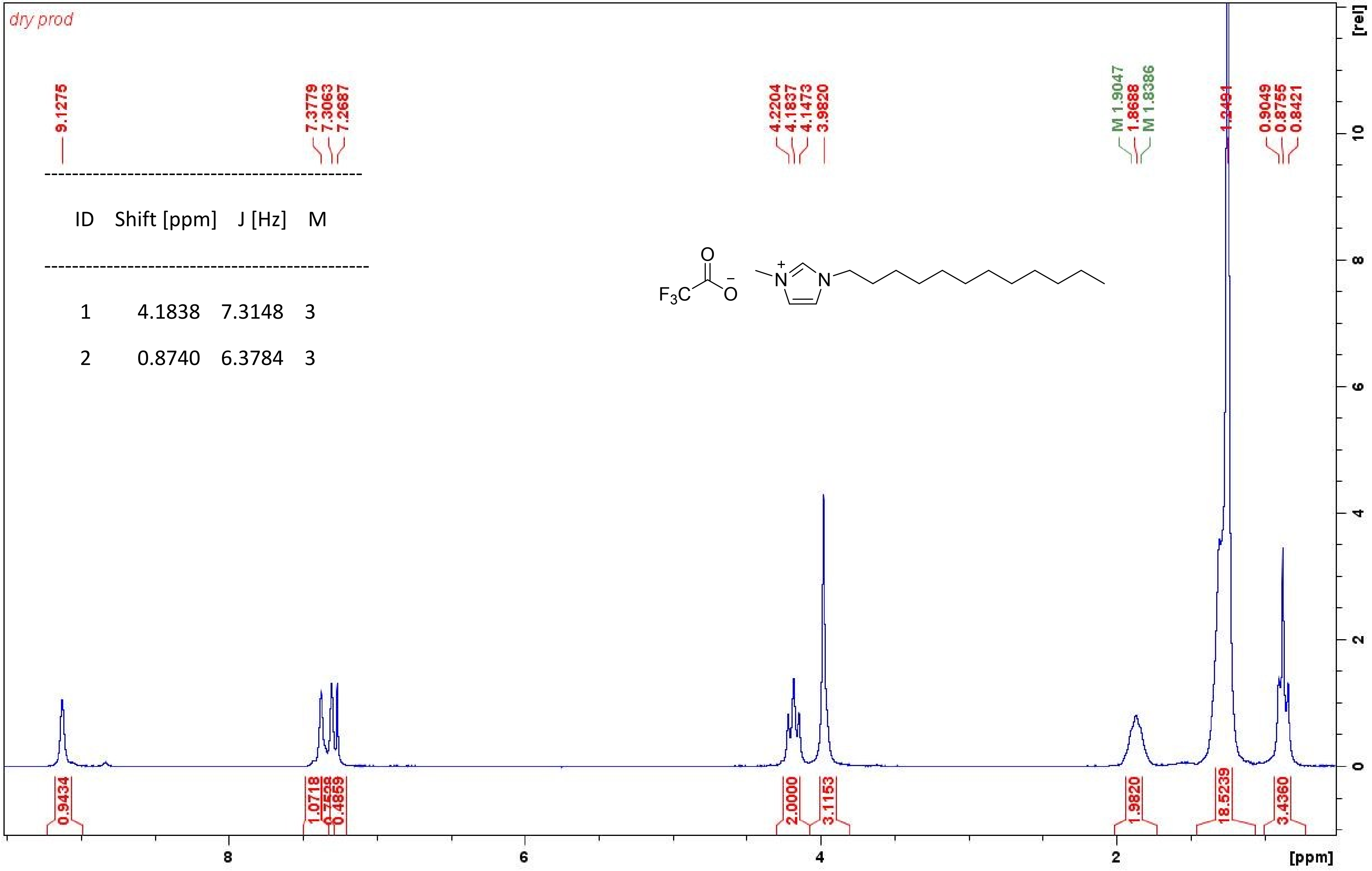
dry prod
9.1275
7.3779
7.3063
7.2687
4.2204
4.1837
4.1473
3.9820
M 1.9047
1.8688
M 1.8386
1.2491
0.9049
0.8755
0.8421
ID Shift [ppm] J [Hz] M
1 4.1838 7.3148 3
2 0.8740 6.3784 3
0.9434
1.0718
2.0000
3.1153
1.9820
18.5239
3.4360
[rel]
[ppm]

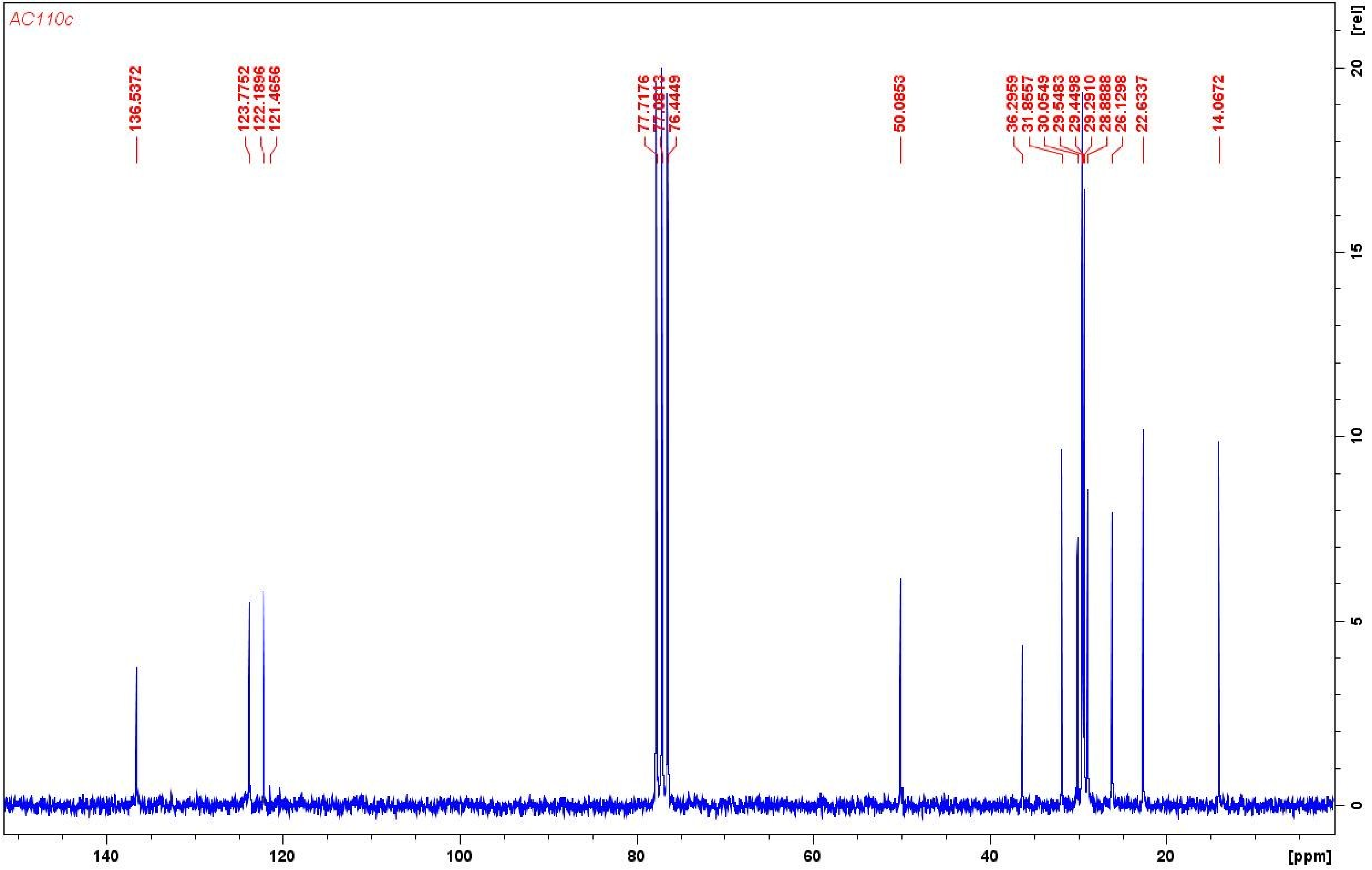
AC110c
136.5372
123.7752
122.1896
121.4656
77.7176
77.0813
76.4449
50.0853
36.2959
31.8557
30.0549
29.5483
29.4498
29.2910
28.8888
26.1298
22.6337
14.0672
[rel]
[ppm]
